# Dynamic Regulation of Granular Hydrogels Through Guest‐Host Interactions to Spatiotemporally Guide Cellular Migration

**DOI:** 10.1002/advs.202512971

**Published:** 2025-11-09

**Authors:** Keisuke Nakamura, Nikolas Di Caprio, Jonathan T. Taasan, Cody O. Crosby, Jason A. Burdick

**Affiliations:** ^1^ BioFrontiers Institute University of Colorado Boulder Boulder CO 80303 USA; ^2^ Department of Chemical and Biological Engineering University of Colorado Boulder Boulder CO 80303 USA; ^3^ Department of Physics Southwestern University Georgetown TX 78626 USA

**Keywords:** 4D bioprinting, granular hydrogels, migration, suspension printing

## Abstract

Cell migration plays a crucial role in the dynamic processes that guide tissue development, regeneration, and repair; yet, developing cell culture platforms that allow control over cell migration in 3D space and time remains a challenge. Here, a strategy is presented using chemically‐responsive granular hydrogels to enable dynamic control over 3D cell migration. Dynamic microgels are fabricated via hyaluronic acid crosslinked via reversible guest–host interactions between adamantane (guest) and β‐cyclodextrin (host), which swell in the presence of a cytocompatible competitive guest molecule (adamantane carboxylic acid, Ad‐COOH) and de‐swell when Ad‐COOH is removed. When formed into granular hydrogels, the addition of Ad‐COOH results in a dynamic porous material with reduced microgel stiffness and increased pore size. Ad‐COOH addition also results in the reduction of mesenchymal stromal cell (MSC) migration from embedded aggregates (spheroids); however, MSC migration returns when Ad‐COOH is removed. Furthermore, suspension bioprinting of jammed spheroids into dynamic granular hydrogels results in 4D printed constructs with patterned cellular regions (e.g., lines, zigzags, spirals) where cellular egress is controlled over time through the presence of Ad‐COOH to create distinct spatiotemporal cellular patterns. This platform offers precise, on‐demand modulation of cell migration, enabling new opportunities to fabricate dynamic, complex engineered tissues.

## Graphical abstract

A dynamic granular system composed of microgels crosslinked through guest‐host interactions is developed to reversibly alter its stiffness and microporosity in response to chemical stimuli, enabling temporal regulation of cell migration. Suspension bioprinting using this dynamic hydrogel allows for 4D spatiotemporal control over tissue formation, demonstrating its potential for unique dynamic cell culture and tissue engineering applications.

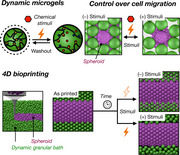

## 1 Introduction

Tissue development and repair are dynamic and complex processes orchestrated by cellular biochemical signaling, as well as cell‐cell and cell‐extracellular matrix (ECM) interactions.^[^ ^1^, ^2^ ^]^ These processes are constantly evolving in response to mechanical and biochemical cues from the surrounding environment, which result in proper tissue formation and function. Cell culture platforms that recapitulate and manipulate these dynamic processes are highly desired for applications in the study of fundamental biology, as well as toward the engineering and repair of tissues.^[^ ^3^, ^4^, ^5^ ^]^

Cell migration is a key step in both tissue development and repair, including embryogenesis, tissue morphogenesis, wound healing, immune surveillance, inflammation, and cancer metastasis.^[^ ^6^, ^7^ ^]^ For example, mesenchymal stromal cells (MSCs) migrate to an injury site, where they locally differentiate or secrete cytokines to promote tissue repair.^[^ ^8^ ^]^ The ECM, along with soluble chemical cues such as growth factors, additionally plays a crucial role in regulating migration processes by spatiotemporally altering biochemical and biophysical signals within the microenvironment.^[^ ^9^, ^10^, ^11^ ^]^ To elucidate migration mechanisms or to direct cell migration for tissue engineering and repair, synthetic biomaterials with tunable properties have been utilized as ECM alternatives.^[^ ^12^, ^13^, ^14^, ^15^, ^16^ ^]^ For example, microfluidic devices have been used to create gradients of chemokines or growth factors, enabling the study of chemotaxis and directional migration.^[^ ^17^, ^18^ ^]^ Hydrogel‐based platforms have also been developed with immobilized or diffusive biochemical gradients to guide collective or single‐cell migration in a controlled manner.^[^ ^19^, ^20^ ^]^ In addition to these biochemical approaches, hydrogels or elastomers with micropatterned stiffness^[^ ^21^, ^22^ ^]^ or geometry,^[^ ^23^, ^24^ ^]^ or those that undergo stimuli‐responsive degradation^[^ ^25^, ^26^ ^]^ or formation,^[^ ^27^ ^]^ have been developed to achieve spatiotemporal control over cell migration. Unfortunately, most existing techniques to control cell migration are limited to simple 2D cell culture systems, and it is challenging to precisely control the timing of migration. Thus, there is a growing need for new technologies that enable spatiotemporal control over 3D cell migration for more sophisticated in vitro models and engineered tissues.

3D bioprinting has enabled the fabrication of cellular constructs through computer‐aided design (CAD) to mimic the complexity of biological tissues and organs, offering great potential for in vitro disease and drug screening models, as well as artificial implants.^[^ ^28^ ^]^ Previous work has explored the use of mechanical extrusion^[^ ^29^, ^30^, ^31^, ^32^, ^33^ ^]^ or photolithography^[^ ^34^, ^35^, ^36^ ^]^ techniques to 3D print cells or spheroids within bioinks (e.g., hydrogels) into spatially organized patterns of heterogeneous cell types. While these techniques can produce organ‐like structures in a static state, the subsequent maturation process of printed cells has not been well understood. To better replicate dynamic tissue processes, there is increasing interest in 4D bioprinting technologies that enable control over cells post printing.^[^ ^37^, ^38^, ^39^, ^40^, ^41^ ^]^ For example, Pramanick et al. reported a 4D bioprinting strategy using support hydrogels in which the shape‐morphing of tissue constructs, driven by cell‐generated forces, was programmed through ECM composition, cell phenotype, and hydrogel viscoelasticity.^[^ ^41^ ^]^ Cell migration in 4D bioprinting is also critical for cell constructs to evolve into more complex patterns. For example, spatially aligned vascularized structures^[^ ^42^ ^]^ and myotube formation^[^ ^43^ ^]^ have been achieved through controlled cell migration after 3D bioprinting with degradable inks or temperature‐responsive polymers. Despite this promise, in most cases, the tissue maturation process that encompasses cell migration remains passive and is largely predetermined by initial conditions such as bioink composition. In contrast, the on‐demand control over migration would be useful to engineer spatially‐organized patterns of cells. In particular, the ability to temporally restrict or promote migration offers a new level of design complexity, enabling printed cells to maintain defined boundaries or to selectively migrate over time.

Granular hydrogels, formed through jammed microgels, are a promising class of materials for 3D bioprinting.^[^ ^15^, ^29^, ^30^, ^40^, ^44^, ^45^, ^46^, ^47^, ^48^, ^49^, ^50^ ^]^ Within these systems, microgels flow under strain by temporarily breaking interparticle contacts, resulting in shear‐thinning and recovery properties. Consequently, granular hydrogels have been used both as bioinks^[^ ^40^, ^47^, ^49^ ^]^ for extrusion‐printing and as support baths^[^ ^29^, ^30^, ^49^, ^51^ ^]^ to stabilize printed structures. Moreover, the porous interstitial space between microgels allows embedded cells to migrate in 3D, which is advantageous for cell delivery and tissue integration.^[^ ^45^, ^48^, ^50^ ^]^ These migration behaviors can also be tuned by tailoring the properties of individual microgels, such as stiffness, size, shape, and bioactivity.^[^ ^15^, ^52^, ^53^, ^54^, ^55^ ^]^ Advances in 4D printing technologies, including those that harness the design landscape of granular materials to actively control cell migration, would open up new opportunities to create more complicated and functional engineered tissues. However, it remains a significant challenge to design dynamic functional hydrogels that modulate biological functions on‐demand while also functioning effectively as bioinks or support baths for 3D/4D printing.

**Figure 1:**
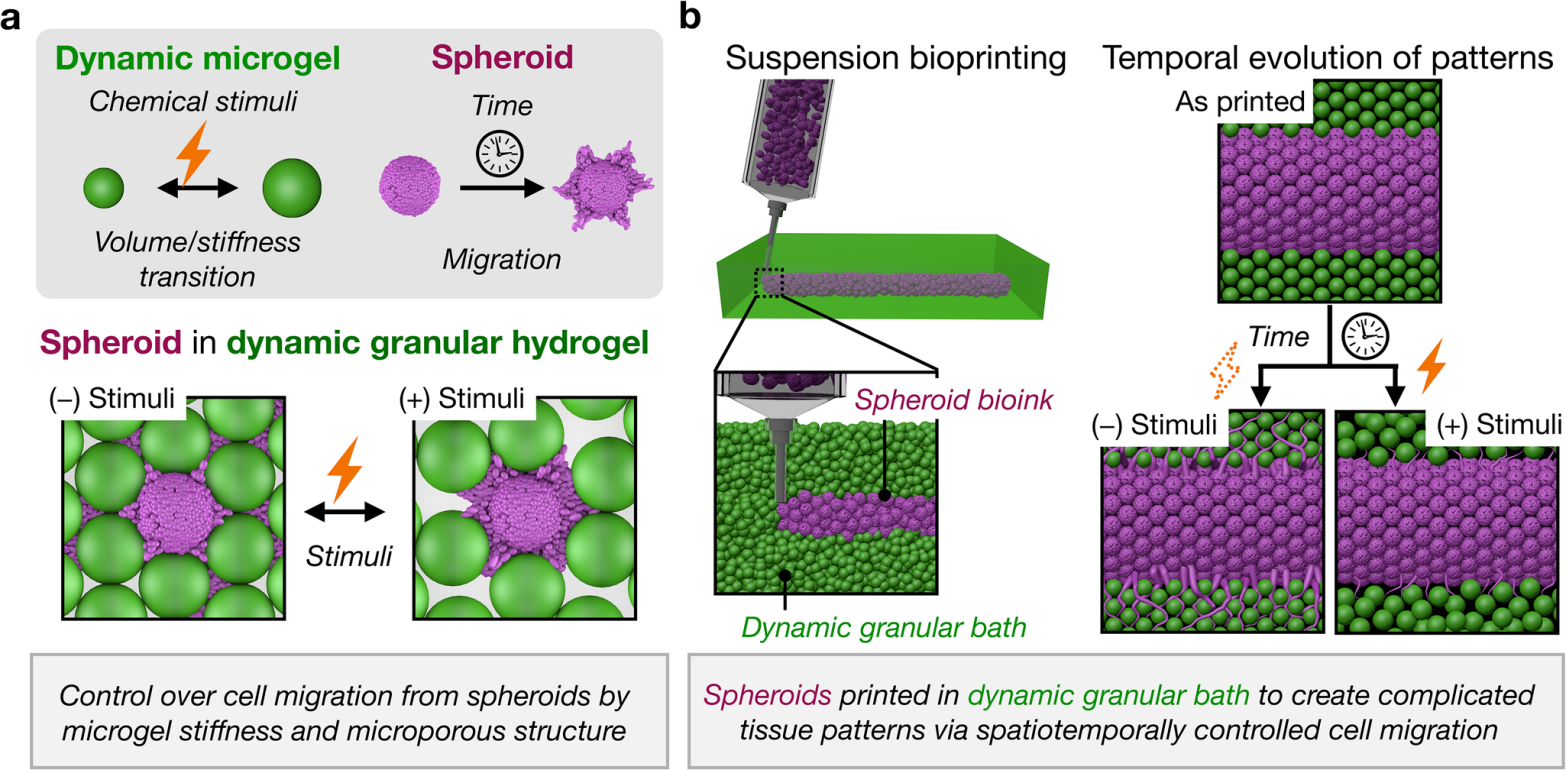
Dynamic granular hydrogels for spatiotemporal control over cellular migration. a) Schematic of dynamic granular hydrogel design to control cell migration. The local microgel properties (e.g., decrease in stiffness) and microporous structure (e.g., increase in porosity) change within the granular hydrogel in the presence of the chemical stimuli. Features such as reduced microgel stiffness and larger pores are expected to reduce cellular migration, enabling temporal control over cellular migration from encapsulated spheroids. b) Schematic of 4D bioprinting to create spatiotemporal patterns of spheroids using suspension bioprinting of spheroid bioinks within dynamic granular hydrogel baths. Printed structures evolve into diverse, complicated patterns over time, depending on the support or suppression of cell migration due to the stimuli‐responsive granular hydrogel.

With this in mind, we hypothesized that the introduction of stimuli‐responsive microgels into granular hydrogels would allow spatiotemporal control over 3D cell migration by altering features of the granular hydrogel, such as microgel mechanics and porosity. To achieve this, we developed microgels based on guest‐host complexes that reversibly swell in response to a chemical stimuli, such as a cytocompatible small molecule that competes with bound guest molecules within the microgel crosslinks (**Figure** **1**a). Upon stimulation, the microgels are expected to increase in volume, resulting in changes in material properties (e.g., decreased microgel stiffness) and changes in granular hydrogel microstructure (e.g., increased pore size and porosity). Based on prior literature, lower microgel stiffness^[^ ^56^ ^]^ and increased porosity^[^ ^14^, ^53^ ^]^ may suppress migration by weakening cell‐matrix interactions and matrix mechanosensing, as well as reducing the continuity of interfaces that support cell egress and migration. Thus, we hypothesized that cells within spheroids embedded in these dynamic granular hydrogels would exhibit reduced migration with stimuli, enabling on‐demand control over cell migration.

MSC spheroids are being widely investigated as therapeutic strategies to heal tissues, such as with bone and cartilage defects; thus, strategies to understand and control MSC migration throughout biomaterials, such as these dynamic granular hydrogel systems, will advance their use in filling defects and repairing tissue. Our lab and others have widely utilized MSC spheroids for tissue repair strategies, but these are most often with static biomaterials.^[^ ^14^, ^57^, ^58^, ^59^, ^60^, ^61^ ^]^ Therefore, in this work, MSC spheroids are used to investigate how cell migration is regulated under dynamic conditions. Furthermore, the 3D printing of MSC spheroids in suspension baths composed of dynamic microgels is implemented to allow for precise spatial patterning of spheroids, which are then temporally controlled (i.e.,.4D printing) via the stimuli‐responsive behavior of the dynamic granular bath (Figure 1b).

## 2 Results and Discussion

### 2.1 Design, Fabrication, and Characterization of Ad‐COOH Responsive Microgels

**Figure 2:**
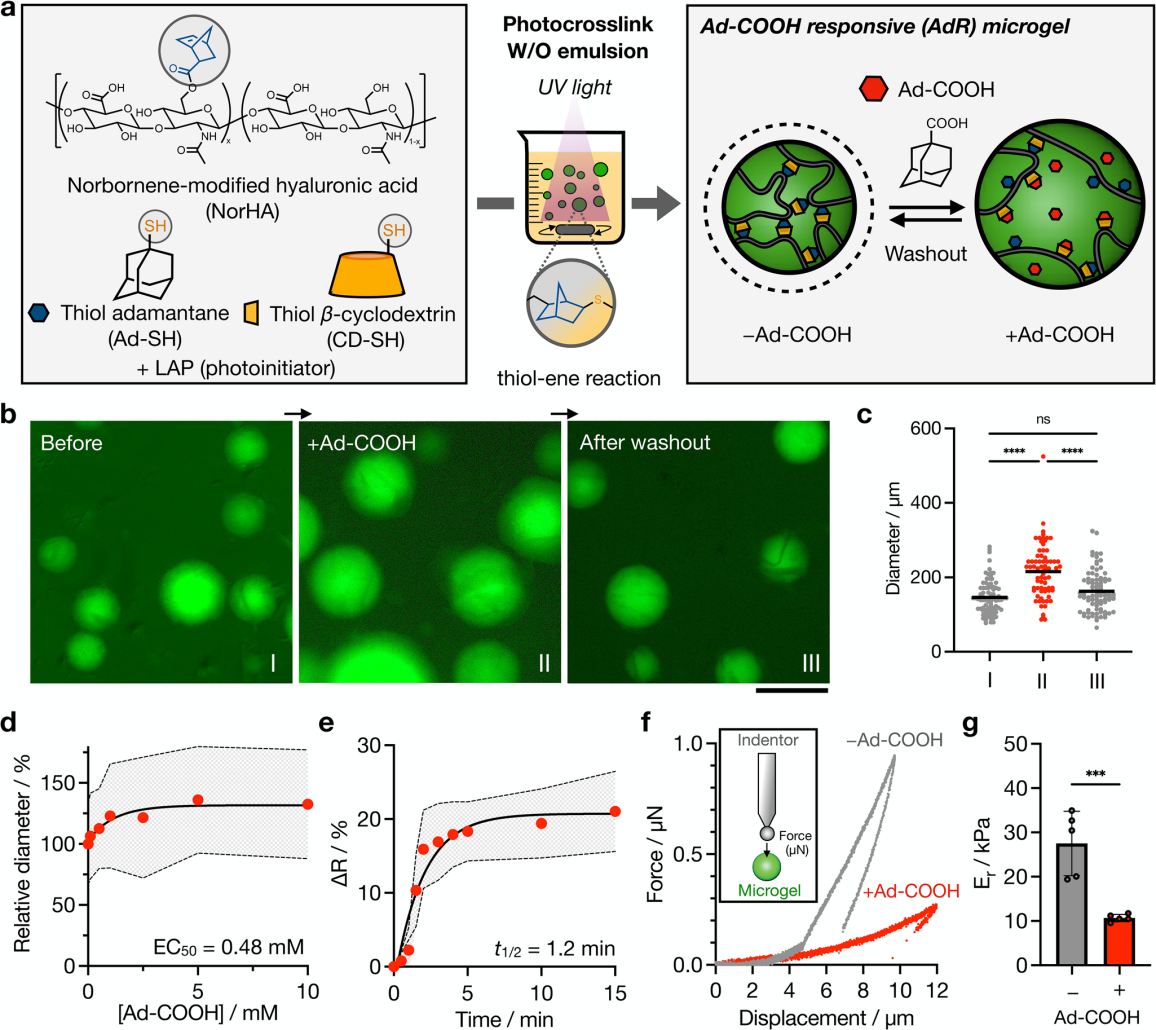
Design, fabrication, and characterization of Ad‐COOH responsive microgels. a) Ad‐COOH responsive (AdR) microgels are fabricated through the thiol‐ene crosslinking of NorHA (3 wt.%) with CD‐SH/Ad‐SH guest‐host (16 mM) complexes in the presence of LAP photoinitiator (0.1 wt.%) via a water‐in‐oil (W/O) batch emulsion and UV light exposure. b) Representative fluorescent images and c) average diameters (mean, *n* = 71 to 86) of AdR microgels before addition of Ad‐COOH (I), in the presence of 5 mM Ad‐COOH (II), and after removal of Ad‐COOH by washing in PBS (III). FITC‐Dextran (0.2 wt.%) is encapsulated in microgels for visualization. Scale bar: 200 µm. d) Relative AdR microgel diameters (*n* = 69 to 113) at varied concentrations of Ad‐COOH (0 to 10 mM) normalized to the diameter without Ad‐COOH. EC_50_: Ad‐COOH concentration that gives a half maximal diameter change. e) Kinetics (half‐life: *t*_1/2_) of Ad‐COOH responsive diameter change after addition of 2.5 mM Ad‐COOH (*n* = 10). f) Representative force versus displacement curves (inset: schematic of spherical indenter and nanoindentation) and g) average reduced modulus (E_r_) values (mean, *n* = 5) of AdR microgels in the absence (Grey) and in the presence (Red) of 5 mM Ad‐COOH. D‐PBS with (+) and without (−) Ad‐COOH is used for all experiments. Data are reported as mean ± standard deviation. ^***^ *p* < 0.001, ^****^ *p* < 0.0001, ns = not significant.

We previously developed dynamic granular hydrogels using temperature‐responsive microgels composed of norbornene‐modified hyaluronic acid (NorHA), crosslinked with temperature‐responsive poly‐N‐isopropylacrylamide linkers.^[^ ^49^ ^]^ Although useful in the design of shape‐morphing materials, the microgels require high temperature (above 45 °C) to induce swelling changes, limiting their applications in the presence of cells. Hippler et al. reported on the development of supramolecular hydrogel microdevices using non‐covalent crosslinking through guest‐host complexes between β‐cyclodextrin (*β*‐CD) and adamantane (Ad) for dynamic mechanical stimulation of cells.^[^ ^62^, ^63^, ^64^ ^]^ The addition of a competitive guest compound, 1‐adamantanecarboxylic acid (Ad‐COOH), dissociates the crosslinks via guest‐host exchange, resulting in swelling and shrinkage of the hydrogels to stretch cells. Inspired by this, we designed Ad‐COOH responsive (AdR) microgels comprised of NorHA crosslinked with *β*‐CD and Ad complexes (**Figure** **2**a). To fabricate microgels, a water‐in‐oil emulsion was first formed through the addition of a solution of NorHA, guest‐host complexes of commercially available thiolated β‐cyclodextrin (CD‐SH) and thiolated adamantane (Ad‐SH), lithium phenyl‐2, 4, 6‐trimethylbenzoylphosphinate (LAP, photoinitiator), and fluorescein isothiocyanate (FITC)‐dextran (for fluorescence imaging) to mineral oil under stirring.^[^ ^65^ ^]^ Droplets were then crosslinked into microgels with UV (ultraviolet) exposure (320–390 nm) via a thiol‐ene reaction, introducing guest‐host complexes as crosslinks to allow swelling with the addition of Ad‐COOH and shrinkage when Ad‐COOH is removed.

To optimize responsiveness, disc‐shaped bulk hydrogels with varying concentrations of NorHA and CD‐SH/Ad‐SH complexes were fabricated to determine which formulation gives the largest swelling response upon addition of Ad‐COOH (Figure S1, Supporting Information). Hydrogels consisting of 3 wt.% NorHA and 16 mM CD‐SH/Ad‐SH complexes exhibited the largest weight change, so this formulation was used to prepare AdR microgels. Based on fluorescent images, the average diameter of the obtained AdR microgels is 146 ± 45 µm (Figure 2b,c). When AdR microgels were placed in the presence of 5 mM Ad‐COOH, their average diameter increased to 216 ± 73 µm. After washing with PBS to remove Ad‐COOH, the average diameter of the microgels returned to a size comparable to the original state (163 ± 54 µm), showing reversibility. To further investigate responsiveness, solutions of various Ad‐COOH concentrations were applied to AdR microgels and their diameters monitored, indicating a plateau at ≈5 mM. The half‐maximal effective concentration (EC_50_) for microgel swelling was determined to be 0.48 mM (Figure 2d). Based on these findings and to ensure cytocompatibility, we used 5 mM Ad‐COOH in the following experiments unless otherwise specified. Kinetics studies reveal that the swelling response is rapid, with a half‐life (*t*_1/2_) of 1.2 min (Figure 2e). Importantly, the hydrogels did not fully dissociate, even with long exposure to Ad‐COOH. This may be attributed to the partial formation of covalent bonds through norbornene‐norbornene reactions, as a side reaction of the thiol‐ene reaction, and due to long light exposures, as reported previously.^[^ ^66^ ^]^

As swelling likely impacts mechanical properties, individual microgels were characterized with spherical probe nanoindentation experiments to obtain their reduced modulus (E_r_) via Oliver Pharr method (Figure 2f,g; Figure S2, Supporting Information). Upon indentation, AdR microgels exhibit E_r_ values of 27.5 ± 7.3 kPa without Ad‐COOH and 10.7 ± 0.8 kPa with 5 mM Ad‐COOH, demonstrating stimuli‐induced softening (Figure 2g). Energy losses are also calculated by subtracting the area under the force‐displacement loading curve from the area of the unloading curve.^[^ ^67^ ^]^ AdR microgels show a higher energy loss without Ad‐COOH than with Ad‐COOH (Figure S2f, Supporting Information), presumably due to stress/strain dissipation through reversible, non‐covalent guest‐host crosslinks.^[^ ^68^ ^]^

**Figure 3:**
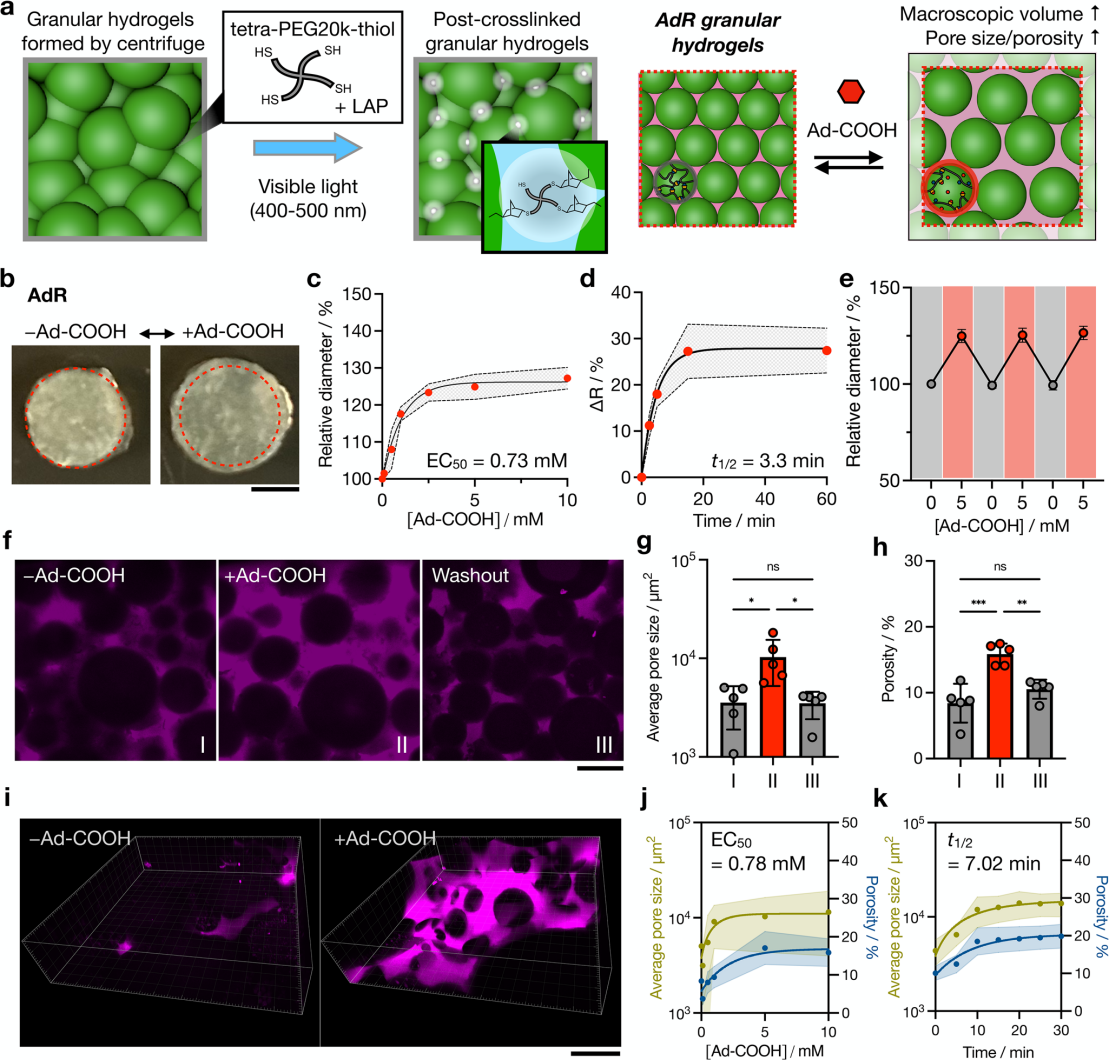
Formation and characterization of dynamic AdR granular hydrogels. a) Schematic of granular hydrogel formation and interparticle post‐crosslinking (left). Microgels are centrifuged (15 000 x g, 5 min) in the presence of 0.1 mM tetra‐PEG20k‐thiol and 0.05 wt.% LAP, followed by visible light exposure (20 mW cm^−2^, 5 min). Schematic of dynamic hierarchical structures of AdR granular hydrogel without and with Ad‐COOH (right). b) Representative photos of post‐crosslinked AdR granular hydrogels treated without (left) and with (right) 5 mM Ad‐COOH. c) Relative AdR granular hydrogel diameters (*n* = 4) at varied concentrations of Ad‐COOH (0 to 10 mM) normalized to the diameter without Ad‐COOH. EC_50_: Ad‐COOH concentration that gives a half maximal diameter change. d) Kinetics (half‐life: *t*_1/2_) of AdR granular hydrogel diameter change after addition of 2.5 mM Ad‐COOH (*n* = 4). e) Relative diameters of AdR granular hydrogels during three repeated cycles of treatment with 5 mM Ad‐COOH (red) and PBS (Grey) (*n* = 4). f to k) Characterization of dynamic microporous structures (TMR‐Dextran (0.1 wt.%) added to visualize void spaces). Representative confocal slices (f) and 3D images (i) of pore structures, g) average pore sizes, and h) average porosities of AdR granular hydrogels without Ad‐COOH (I), in the presence of 5 mM Ad‐COOH (II), and after removing Ad‐COOH by washing in PBS (III), *n* = 5. Scale bar: 200 µm. j) Average pore sizes (Green) and average porosities (Blue) of AdR granular hydrogels at varied concentrations of Ad‐COOH (0 to 10 mM) normalized to the diameter without Ad‐COOH (*n* = 5). k) Kinetics of change in average pore size (Green) and average porosity (Blue) of AdR granular hydrogels after addition of 5 mM Ad‐COOH (*n* = 5). D‐PBS with (+) and without (‐) Ad‐COOH is used for all experiments. Data are reported as mean ± standard deviation. ^*^ *p* < 0.05, ^**^ *p* < 0.01, ^***^ *p* < 0.001, ns = not significant.

For control studies, non‐responsive (NR) microgels were fabricated in the same manner, but using dithiothreitol (DTT) as the crosslinker instead of the guest‐host complexes (Figure S3a, Supporting Information). Two NR microgel groups were prepared using 4 or 8 mM DTT. Microgels crosslinked with 8 mM DTT (stiff NR microgels) have an average diameter of 150 ± 64 µm and an E_r_ value of 23.0 ± 2.7 kPa, matching the AdR microgels in the absence of Ad‐COOH (Figures S2e and S3b, Supporting Information). On the other hand, microgels crosslinked with 4 mM DTT (soft NR microgels) have the same diameter (150 ± 64 µm) but a lower E_r_ of 9.5 ± 1.1 kPa, which is comparable to that of AdR microgels when treated with 5 mM Ad‐COOH (Figures S2e and S3b, Supporting Information). Both NR microgel groups show no change in their average diameter with the addition of Ad‐COOH, confirming that the response of AdR microgels is due to dissociation of the CD/Ad complexes (Figure S3b, Supporting Information).

Overall, this work indicates successful fabrication of chemically‐responsive AdR microgels that reversibly tune their size and stiffness in response to Ad‐COOH.

### 2.2 Preparation and Characterization of Ad‐COOH Responsive Granular Hydrogels

With AdR microgels in hand, we next investigated the properties of granular hydrogels formed by the centrifugation and crosslinking of AdR microgels. Post‐crosslinking is essential as dynamic granular hydrogels are likely to fall apart during stimuli response if only jamming is used.^[^ ^49^ ^]^ Specifically, tetra‐PEG20k‐thiol and LAP were introduced into the interstitial space, and visible light irradiation was used to link the microgels together covalently through the thiol‐ene reaction between the thiol groups of tetra‐PEG20k‐thiol and residual norbornene groups on the microgels (**Figure** **3**a). After post‐crosslinking into discs, the granular hydrogels maintained their shape even after removal from the mold and then exhibited changes in their disc diameter with the addition of Ad‐COOH (Figure 3b). Specifically, the bulk responsiveness of the AdR granular hydrogels is shown by an Ad‐COOH concentration‐dependent increase in diameter up to ≈30% with an EC_50_ of 0.73 mM, which is comparable to the response of individual AdR microgels in a suspended state (Figure 3c). The kinetics for Ad‐COOH induced swelling are as rapid as suspended microgels, and the response is complete within 15 min (Figure 3d). This response is reversible, and the swelling/shrinking cycles were repeated at least three times without noticeable change in the response (Figure 3e).

In addition to the bulk response, we also explored the microporous structures of AdR granular hydrogels. Confocal laser scanning microscopy (CLSM) imaging was conducted to visualize interstitial void spaces by incorporating tetramethylrhodamine (TMR)‐Dextran, which preferentially diffuses through pores but not into microgels. Z‐stacked images were obtained, and each slice was analyzed to quantify average pore size and porosity, as previously described.^[^ ^69^ ^]^ Before the addition of Ad‐COOH, the average pore size and porosity were 3553 ± 1661 µm^2^ and 8.4 ± 2.9%, respectively (Figure 3f–h). Upon treatment with 5 mM Ad‐COOH, microgels swell, which results in increases in both pore size (10 307 ± 5076 µm^2^) and porosity (15.9 ± 1.6%). After removal of Ad‐COOH by washing in PBS, these parameters return to values close to the original measurements (3052 ± 1086 µm^2^ and 10.5 ± 1.4%), demonstrating reversibility. 3D reconstructions of z‐stacked images show that these pores are interconnected and that structural changes occur in three dimensions (Figure 3i). Moreover, both pore size and porosity can be finely tuned by varying Ad‐COOH concentration (Figure 3j), and the concentration dependence and kinetics of microporous changes are comparable to those of the bulk response (Figure 3j,k). Time‐lapse images of this swelling process suggest the partial loss of interparticle contacts as the microstructure changes, which contributes to overall changes in the granular hydrogel with the addition of the Ad‐COOH (Figure S4, Supporting Information). These findings suggest that microgels swell cooperatively and isotopically, leading to an increase in porosity and macroscopic volume in the AdR granular hydrogel (Figure 3a). The combined results demonstrate that AdR granular hydrogels can be dynamically tuned with regard to both macroscopic volume and microporous structure by Ad‐COOH addition and removal.

Additionally, we investigated the response of AdR granular hydrogels when exposed to Ad‐COOH under confined conditions (i.e., remain in mold, rather than removed from the mold as in the studies above). Specifically, AdR granular hydrogels were post‐crosslinked within a mold and directly treated with Ad‐COOH in situ (Figure S5a, Supporting Information). Under confined conditions, neither the pore size or the porosity significantly changed with the addition of Ad‐COOH (Figure S5b,c, Supporting Information). This observation is consistent with previous reports that microgel swelling can be suppressed by physical confinement.^[^ ^70^, ^71^ ^]^ Likewise, in our system, the responsiveness of AdR microgels to Ad‐COOH was diminished under confined conditions, suggesting that a free‐floating, unconfined environment is essential to achieve bulk and microporous dynamic response in response to Ad‐COOH.

### 2.3 Control Over Cell Migration from bMSC Spheroids Embedded in Granular Hydrogels

**Figure 4:**
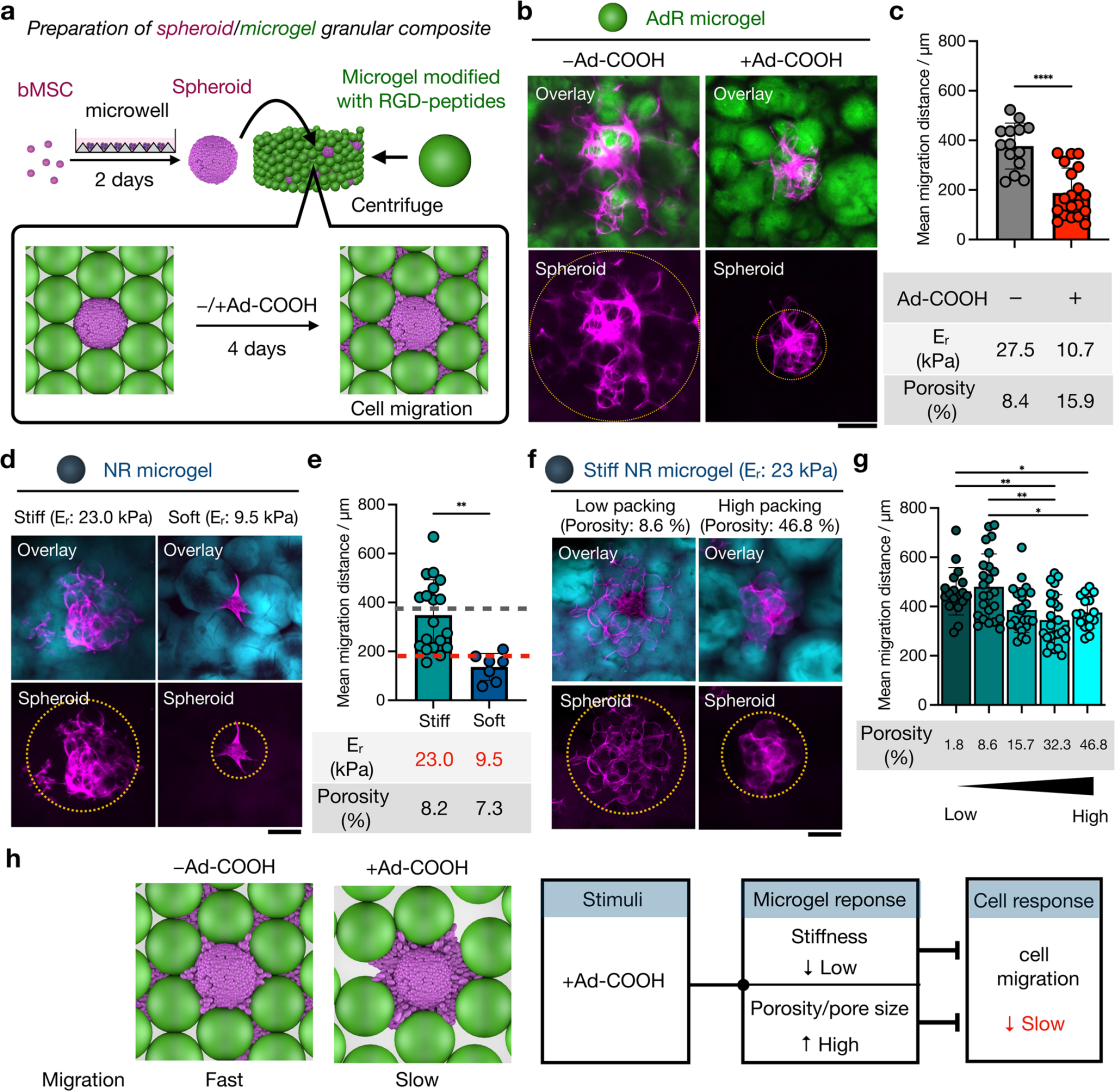
Migration of bMSCs within AdR granular hydrogels. a) Schematic of granular hydrogel composite preparation through the mixing of bovine mesenchymal stromal cell (bMSC) spheroids with microgels modified with RGD‐peptide (2 mM) and post‐crosslinking. bMSC (P3, 1000 per well) seeding on pyramidal PDMS molds is used to form spheroids over 2 days. Spheroids are cultured in media with and without Ad‐COOH for 4 days, fixed, and imaged by confocal microscopy. b) Representative z‐projection confocal images and c) mean migration distances of bMSCs in the AdR granular hydrogel without (left, *n* = 14) and with (right, *n* = 19) 5 mM Ad‐COOH. Magenta: spheroids (Phalloidin‐Alexa647, actin), Green: AdR microgel (FITC‐Dextran). Scale bar: 200 µm. E_r_ and average porosity at each condition are also shown. d to g) Mechanistic studies on cell migration using non‐responsive (NR) granular hydrogels. d) Representative z‐projection images and e) mean migration distances of bMSCs in NR granular hydrogels with different stiffnesses (Soft: 9.5 kDa, Stiff: 23 kPa), *n* = 7 or 19. Cyan: NR microgel (FITC‐Dextran). Migration distances in AdR granular hydrogels without (Grey) and with Ad‐COOH (Red) are shown as dashed lines for comparison. f) Representative z‐projection images and g) mean migration distances of bMSCs in stiff NR granular hydrogels with various packing densities, *n* = 17 to 25. h) Schematic of cell migration behavior without and with Ad‐COOH, where the presence of Ad‐COOH decreases microgel stiffness and increases granular hydrogel porosity, which both contribute to the suppression of cell migration. Data are reported as mean ± standard deviation. ^*^ *p* < 0.05, ^**^ *p* < 0.01. ^****^ *p* < 0.0001, ns = not significant.

Spheroids offer a defined starting point for cell migration, and spheroid‐based assays have been used to quantitatively analyze cell movement within 3D matrices, providing insight into how material properties influence cell migration.^[^ ^14^, ^72^ ^]^ Here, we aggregated juvenile bovine bone marrow mesenchymal stromal cells (bMSCs) into spheroids (pyramidal polydimethylsiloxane (PDMS) molds, average diameter of 170 ± 19 µm (Figure S6, Supporting Information)), embedded them in AdR granular hydrogels with visible light post‐crosslinking, and monitored bMSC migration from the spheroids with and without stimuli over 4 days (**Figure** **4**a). To enable bMSC adhesion to microgels, thiol‐containing RGD peptides (sequence: GCGYGRGDSPG) were covalently linked within the NorHA network via thiol‐ene reaction during microgel preparation. Actin staining and confocal imaging were used to measure and report migration distance.^[^ ^15^, ^69^ ^]^ After 4 days of culture without Ad‐COOH, bMSCs attached onto the microgel surface and migrated between the microgels outward from the spheroid, with a migration distance of 377 ± 93 µm (Figure 4b,c). In contrast, in the presence of Ad‐COOH, cell migration was suppressed, with a migration distance of 181 ± 101 µm. These results demonstrate that bMSCs exhibit differential migration within AdR granular hydrogels depending on the presence of Ad‐COOH. Notably, when spheroids were encapsulated in bulk AdR granular hydrogels, MSCs did not migrate after 4 days, even without Ad‐COOH, demonstrating that the microporous structures of the granular hydrogel is crucial for 3D migration (Figure S7, Supporting Information).

To further understand why migration is reduced in AdR granular hydrogels with Ad‐COOH, but not without Ad‐COOH, we also investigated bMSC migration in non‐responsive (NR) granular hydrogels, formed from microgels with a non‐responsive crosslinker. NR granular hydrogels formed from RGD‐modified hydrogels with high and low crosslink densities exhibit differential E_r_ (23.0 ± 2.7 kPa for “stiff” v.s. 9.5 ± 1.1 kPa for “soft”; Figure S2, Supporting Information) that are similar to the E_r_ of microgels in the absence and presence of Ad‐COOH, respectively. Interestingly, post‐crosslinked granular hydrogels of the RGD‐modified soft and stiff NR microgels both have pore sizes and porosity comparable to those of AdR granular hydrogels without Ad‐COOH present (Figure S8, Supporting Information). Thus, these groups allow investigation of how the combined influence of microgel mechanics and pore structure influences bMSC migration.

In stiff NR granular hydrogels, the migration distance is comparable to that in AdR microgels without Ad‐COOH (348 ± 144 µm), which are formed from microgels with similar mechanics (Figure 4d,e). Notably, the migration of bMSCs from spheroids in stiff NR granular hydrogels shows no significant difference between conditions with and without Ad‐COOH (Figure S9, Supporting Information), suggesting that Ad‐COOH itself does not affect cell migration but rather changes in cell migration are due to altered material properties of AdR granular hydrogels. Cell migration is reduced in soft NR granular hydrogels (136 ± 55 µm) compared to stiff NR granular hydrogels (348 ± 144 µm) (Figure 4d,e). Since both have similar pore sizes and porosities, this differential cell migration is likely due to differences in E_r_ (stiff: 23.0 ± 2.7 kPa, soft: 9.5 ± 1.1 kPa; Figure S2e, Supporting Information). Further, the migration of bMSCs in AdR granular hydrogels treated with Ad‐COOH matches the soft NR granular hydrogels (Figure 4e), suggesting that microgel stiffness may be one of the key factors controlling bMSC migration, as these groups also had similar mechanical properties.

Beyond microgel stiffness, as shown above, porosity changes in AdR granular hydrogels with and without Ad‐COOH; thus, it is important to investigate the effect of porosity on migration in stable materials. Using stiff NR granular hydrogels, five different granular hydrogels were prepared (varied centrifugation speeds or dilutions (Figure S10a, Supporting Information)) having porosities of 8.6% to 48% and pore sizes of 691 to 173 219 µm^2^ (two representative conditions shown in Figure 4f; remaining shown in Figure S10b,c, Supporting Information). The migration distances with varied porosities/pore sizes decrease slightly as porosities/pore sizes increase (Figure 4g; Figure S10d, Supporting Information). This is presumably because the hydrogels with higher packing density present larger surface‐area‐to‐volumes, providing more surfaces for cell attachment and overall confinement to promote migration, as previously reported in several porous hydrogel systems.^[^ ^14^, ^53^ ^]^ These results suggest that increased porosity and pore size in AdR granular hydrogels with Ad‐COOH contribute to reduced migration.

Taken together, the addition of Ad‐COOH decreases microgel stiffness and increases granular porosity/pore size, which may both contribute to the reduction in cell migration (Figure 4h). Migration is governed by a combination of numerous biochemical and biophysical factors, which introduces complexity into this behavior. For example, in addition to stiffness, the viscoelastic properties of microgels may also influence cell migration, which has been reported by others.^[^ ^73^, ^74^ ^]^ As described above, AdR microgels reduce their stiffness upon the addition of Ad‐COOH, but there is evidence that this also decreases microgel viscoelasticity (Figure S2f, Supporting Information). This decrease in viscoelasticity might also contribute to reduced migration. In addition, the density of adhesive peptides changes as microgels swell, and the decreased concentration of RGD peptide may also suppress migration.^[^ ^75^, ^76^, ^77^ ^]^ In our design, the surface area of the microgels increases ≈85%, meaning that there will be a similar reduction in RGD density, which is fairly modest. Despite the complexity, it is clear that migration is controlled through the addition of the small molecule Ad‐COOH within our engineered platform.

### 2.4 Temporal Control of bMSC Migration Under Dynamic Cell Culture Conditions

**Figure 5:**
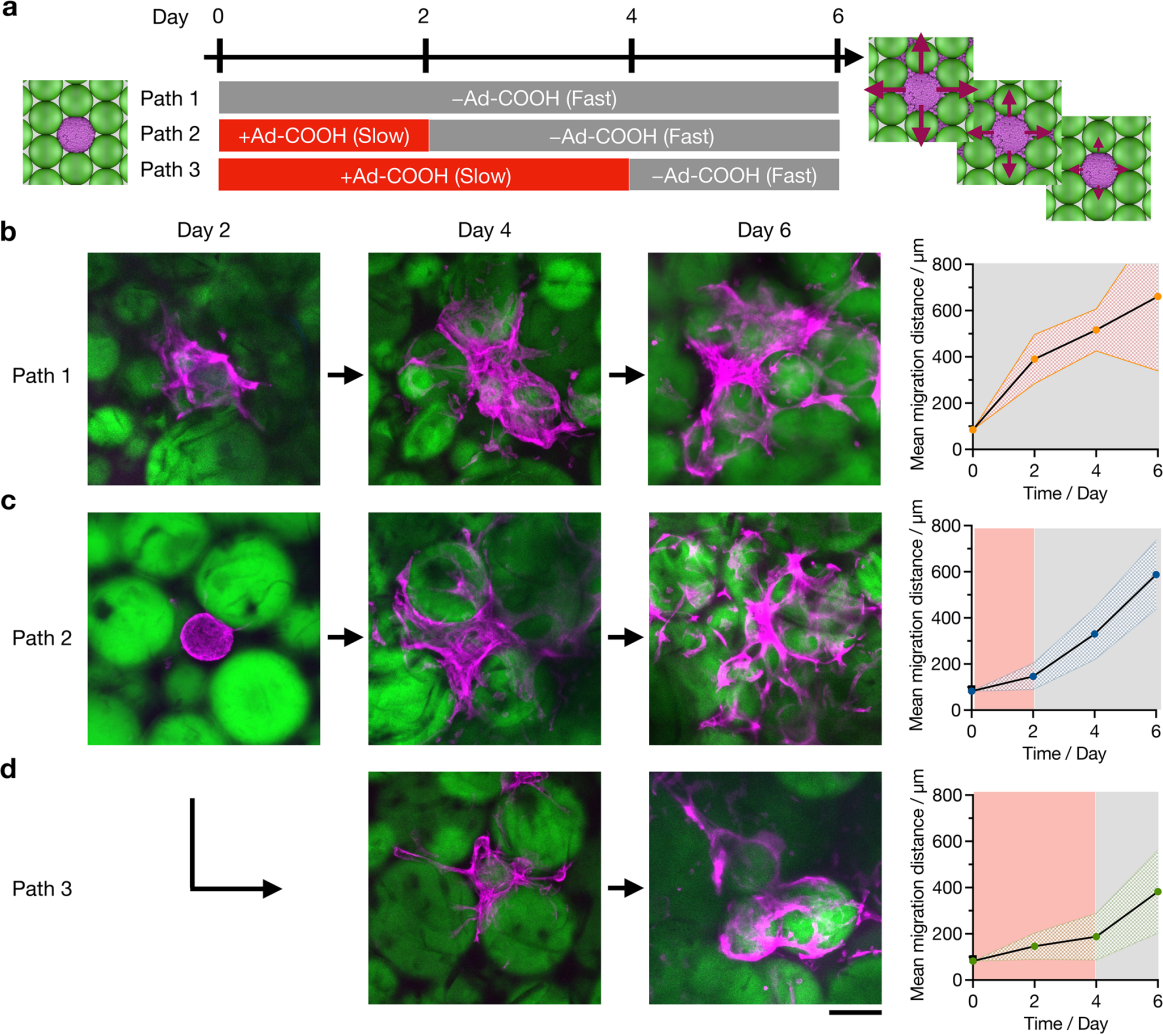
Dynamic control of bMSC migration in AdR granular hydrogels. a) Experimental design for the temporal control of bMSC migration by changing Ad‐COOH during culture. Ad‐COOH in culture media is switched from 5 mM to 0 mM at day 0 (path 1), day 2 (path 2), or day 4 (path 3). b to d) Representative z‐projection images of bMSC migration from spheroids in Ad granular hydrogels at day 2, day 4, and day 6 in different paths. b) path 1, c) path 2, d) path 3. Mean migration distances at different time points (0, 2, 4, 6 days) are plotted on the right of images of each condition (Red shading +Ad‐COOH; Grey shading –Ad‐COOH), *n* = 7 to 19. Magenta: spheroids (Phalloidin‐Alexa647, actin), Green: AdR microgel (FITC‐Dextran). Scale bar: 200 µm. Data are reported as mean ± standard deviation.

Considering the varied cell migration in AdR granular hydrogels in the presence or absence of Ad‐COOH, we next explored the potential to temporally control cell migration under dynamic cell culture conditions. Specifically, we investigated whether the migration of bMSCs from spheroids is enhanced on‐demand by replacing cell culture media containing 5 mM Ad‐COOH (where migration is low) with media free of Ad‐COOH (where migration is high) at different time points: day 0 (path 1), day 2 (path 2), day 4 (path 3) and quantifying migration over time (**Figure** **5**a). In path 1, cells from spheroids exhibit the greatest migration over the 6‐day period, resulting in a migration distance of 661 ± 322 µm) by day 6 (Figure 5b). In path 2, migration is initially low at day 2 (122 ± 57 µm), which is lower than path 1 (390 ± 106 µm); however, following the media exchange, bMSC migration increases to reach a greater distance by day 6 (588 ± 149 µm) (Figure 5c). In path 3, migration remains suppressed until day 4, ultimately resulting in the lowest overall migration distance among all conditions at day 6 (383 ± 180 µm) (Figure 5d). The migration distance in this case was slightly higher than that treated with Ad‐COOH for the entire 6 days (287 ± 99 µm), suggesting that migration can still be switched even at day 4 (Figure S11, Supporting Information). These results are intriguing, as the increase in migration on‐demand is unique. Again, this is a complex combination of numerous features that change in the presence and absence of Ad‐COOH, but the findings clearly demonstrate that AdR granular hydrogels allow dynamic control of cell migration with changes in culture conditions and resulting material properties.

### 2.5 Suspension 4D Bioprinting of bMSC Spheroids in AdR Granular Baths to Control Spatiotemporal Cellular Patterns

**Figure 6:**
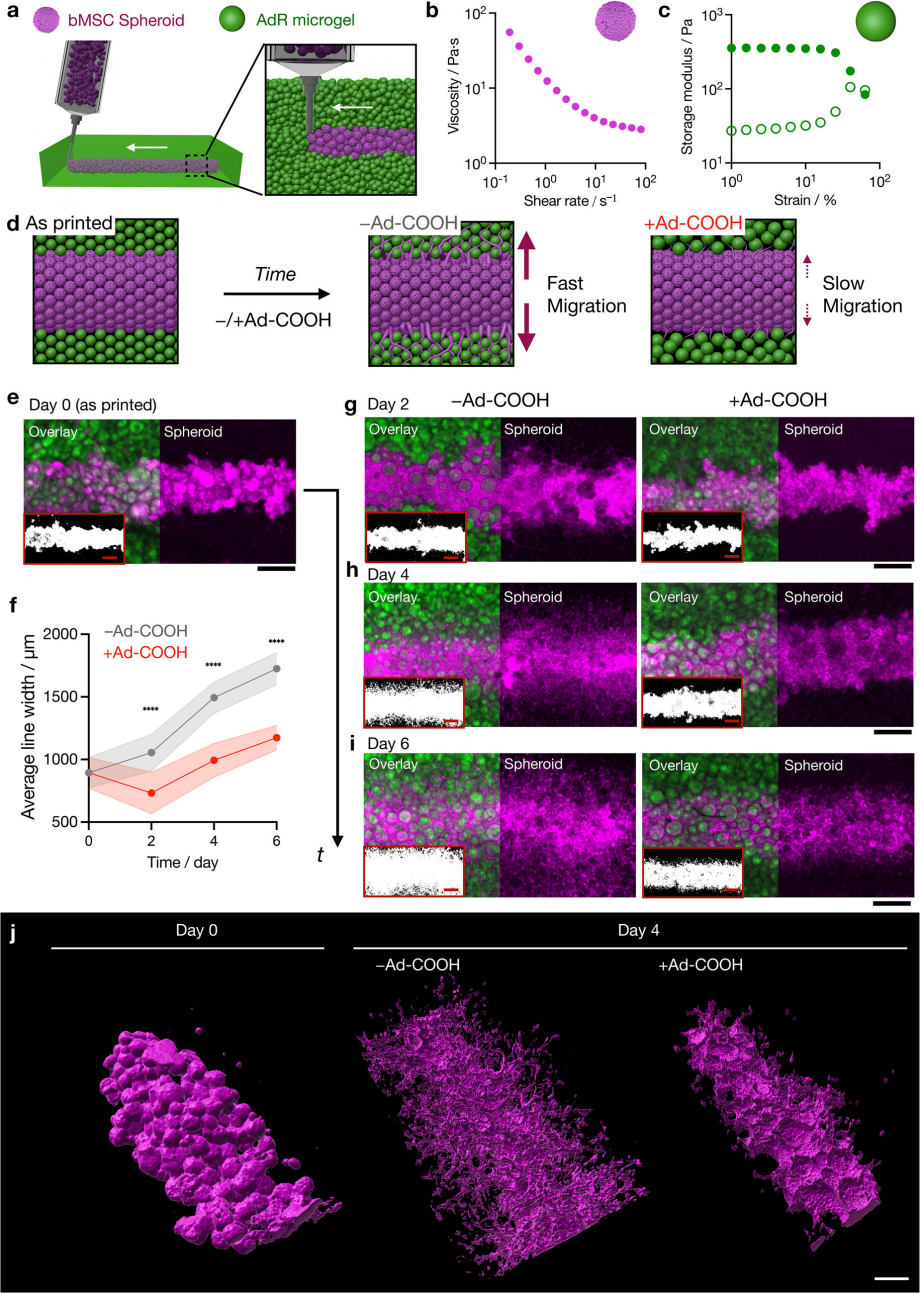
4D bioprinting of bMSC spheroids in an AdR granular bath. a) Schematic of suspension printing of jammed bMSC spheroids in an AdR granular suspension bath. The printed structures are post‐crosslinked by visible light exposure. b) Viscosity versus shear rate for jammed spheroids. c) Strain sweep rheology for AdR granular hydrogels. Storage (G', filled) and loss (G””, open) moduli; Frequency: 1 rad s^−1^. d) Schematic of the temporal change of printed lines of spheroids in constructs with and without Ad‐COOH. e, g, h, i) Representative z‐projection confocal images (left: without Ad‐COOH; right: with 5 mM Ad‐COOH) of printed lines at day 0 (e), day 2 (g), day 4 (h), and day 6 (i). Magenta: spheroids (Phalloidin‐Alexa647, actin), Green: AdR microgel (FITC‐Dextran). Binarized images of cells are also shown as insert images. Scale bar: 500 µm. (f) Average width (*n* = 15) of printed lines at different time points in the absence (Grey) or the presence (Red) of Ad‐COOH. ^****^ *p* < 0.0001. Data are reported as mean ± standard deviation. j) 3D surface rendered images of spheroids at day 0 (left) and at day 4 without (middle) and with (right) Ad‐COOH. Scale bar: 200 µm.

Beyond a model of cell migration, spheroids exhibit high cell densities with inherent cell‐cell interactions and a potent secretome, which are desirable to mimic the native tissue developmental process.^[^ ^78^, ^79^ ^]^ Further, numerous studies have explored the use of spheroids as building blocks to fabricate high cell density tissues. As an example, suspension bath printing has been employed to support spheroid constructs and help spheroids fuse into larger, continuous tissue structures,^[^ ^80^ ^]^ particularly due to the shear‐thinning and recovery properties of both granular materials and jammed spheroids.^[^ ^48^, ^81^ ^]^ Here, we used AdR granular hydrogels as a suspension bath to print spheroid inks that can be subsequently stabilized with light, and used their dynamic properties to regulate cell migration as a 4D bioprinting approach (**Figure** **6**a). First, we illustrate the shear‐thinning and recovery properties of bMSC spheroid inks, which allow the jammed spheroids to flow through a needle during extrusion and immediately stabilize after extrusion (Figure 6b; Figure S12, Supporting Information). Similarly, AdR granular hydrogels exhibit shear‐yielding and recovery properties, allowing them to deform with a needle to receive a printed bioink and then return to their original state, properties important for use as a suspension bath (Figure 6c; Figure S12, Supporting Information).

Jammed bMSC spheroids were then directly 3D printed into a rectangular AdR granular bath (8 mm x 6 mm x 1.5 mm) to create 6‐mm line patterns using a custom‐built 3D printer equipped with a modified extruder (Figure S13, Supporting Information). After visible light‐mediated post‐crosslinking, the printed objects were cultured in the presence and absence of 5 mM Ad‐COOH to investigate temporal changes in the spheroids over time (Figure 6d). Immediately after printing (day 0), spheroids were spatially patterned in lines within the AdR granular bath (Figure 6e), with an initial average line width of 893 ± 123 µm (Figure 6f). Sliced images show that spheroids and surrounding microgels are primarily spatially segregated across dimensions, suggesting that the spheroid ink can be stabilized immediately after extrusion (Figure S14, Supporting Information). Over time (day 2, day 4, day 6), the pattern evolves differently depending on the presence of Ad‐COOH. At day 2, no drastic differences were observed between the two conditions (Figure 6g); however, by day 4 and especially day 6, substantial differences in the morphologies emerge. In the absence of Ad‐COOH, bMSCs migrated outward from the printed spheroids along microgel surfaces, forming branched structures and increasing the width of the cell distribution to 1724 ± 129 µm by day 6 (Figure 6f,i,h). In contrast, with Ad‐COOH, cells remained largely within the printed regions, resulting in less branched morphologies and narrower line widths (1173 ± 98 µm at day 6) (Figure 6i).

To further evaluate the 3D morphology of printed structures, z‐stacked images were processed into 3D surface‐rendered reconstructions (Figure 6j; Figure S15 and Movies S1–S3, Supporting Information). At day 0, the printed lines consisted of non‐fused spheroids; however, by day 2 and 4, the spheroids within the lines fused and were more continuous in both conditions. Furthermore, the constructs cultured without Ad‐COOH exhibited more extensive branching and a greater number of protrusions in 3D when compared to those cultured with Ad‐COOH. These results suggest that both spheroid fusion and cell migration along the microgel surfaces contribute to the tissue maturation process. Importantly, the suppression of cell migration in the presence of Ad‐COOH results in distinct spatiotemporal morphologies. By leveraging the dynamic properties of granular hydrogels, this platform offers a versatile approach to create complex, spatiotemporally patterned tissue constructs.

**Figure 7:**
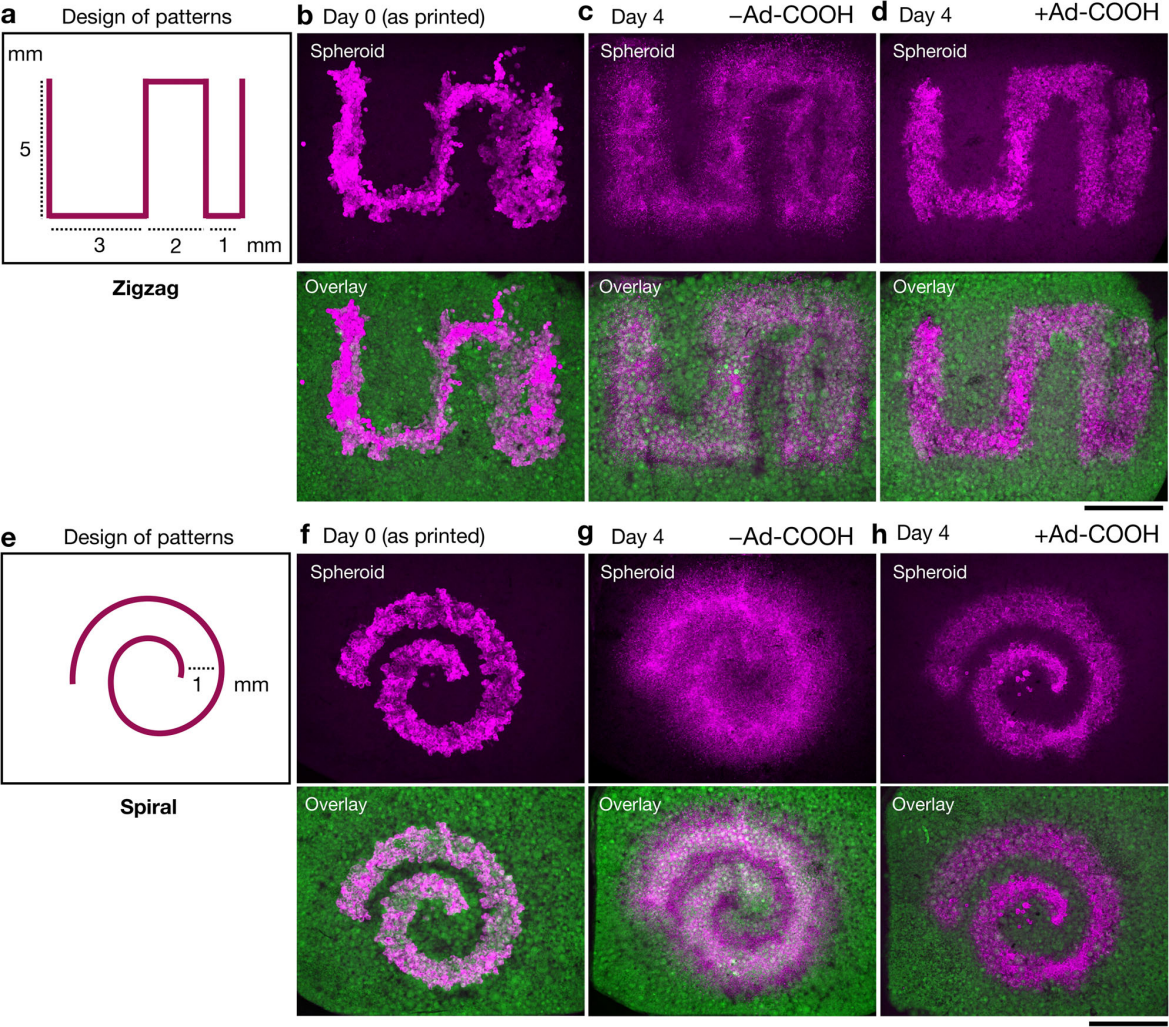
4D migration patterns from spheroids, based on 3D printed patterns. a) Design of zigzag pattern, where the spacing between lines changes. b, c, d) z‐projection confocal images (b: day 0; c: day 4 without Ad‐COOH; d: day 4 with 5 mM Ad‐COOH) of zigzag‐shaped lines (shown as top: spheroids alone, bottom: overlay of spheroids and microgels). e) Design of spiral pattern. f, g, d) z‐projection confocal images (f: day 0; g: day 4 without Ad‐COOH; h: day 4 with 5 mM Ad‐COOH) of spiral‐shaped lines (shown as top: spheroids alone, bottom: overlay of spheroids and microgels). Magenta: spheroids (Phalloidin‐Alexa647, actin), Green: AdR microgel (FITC‐Dextran). Scale bar: 2 mm.

Finally, to highlight the advantages of this dynamic granular hydrogel system for 3D bioprinting, we programmed distinct spatial patterns of spheroids during the printing process (**Figure** **7**). Zigzag‐ and spiral‐shaped patterns of bMCS spheroids were successfully printed with good fidelity within the dynamic granular bath (Figure 7b,f). Following 3D printing, these constructs exhibited distinct pattern evolution by controlling the extent of migration based on the presence or absence of Ad‐COOH (Figure 7c,d,g,h). Notably, with Ad‐COOH, distinct cellular regions were retained over time, whereas without Ad‐COOH, cells migrated between the printed regions. These results demonstrate the utility of our approach for fabricating more complex, tissue‐like structures by combining the precise spatial patterning of 3D printing with the temporal regulation of cellular migration via chemical stimuli. Further work could adapt to printing more complex materials as well, which could even be used to guide the directionality of cell migration by spatially changing these signals across cell aggregates or by introducing alternate chemotactic signals.

## 3 Conclusion and Outlook

In this study, we developed a unique dynamic granular hydrogel that enables spatiotemporal control of bMSC migration in 3D through the simple addition or subtraction of a small molecule, Ad‐COOH. This molecule disrupts crosslinks within microgels crosslinked via guest‐host interactions to increase microgel swelling, resulting in a decrease in microgel mechanics and an increase in granular hydrogel microporosity. When washed out, microgels respond accordingly with a decrease in swelling, which increases microgel mechanics and reduces granular hydrogel microporosity. Changes in these properties combine to alter the migration of bMSCs from embedded spheroids, including temporally. Furthermore, suspension printing of spheroid bioinks into dynamic AdR granular baths allowed the fabrication of 4D cellular constructs that evolve into distinct spatial tissue patterns through temporal regulation of cell migration. While reversible host–guest chemistry has been studied in bulk responsive hydrogels,^[^ ^62^, ^63^, ^64^, ^82^ ^]^ the responsive behavior and biointerface properties of dynamic granular hydrogels remain largely unexplored. Our findings offer new insights and design principles for such dynamic materials, with potential applications in bioprinting and tissue engineering, where controlled migration can be used in the design of in vitro platforms to investigate biological pathways in development or in the guiding of tissue formation in 3D systems based on printed patterns of cellular aggregates, as well as in applications such as soft robotics,^[^ ^49^ ^]^ sensing devices,^[^ ^71^ ^]^ and agriculture.^[^ ^83^ ^]^

Existing dynamic cell culture platforms have primarily employed stimuli‐responsive materials based on light and temperature, such as photo‐degradable^[^ ^25^ ^]^ and photo‐crosslinkable hydrogels,^[^ ^27^ ^]^ to regulate cell migration by altering physical confinement within bulk matrices. However, conventional stiffening/softening strategies based on bulk stimuli‐responsive hydrogels often face challenges when integrated into bioprinting, which demands strict requirements in rheological properties, crosslinking kinetics, and cytocompatibility. In contrast, our granular hydrogel system exhibits inherent shear‐thinning behavior, injectability, and modularity, making it highly compatible with extrusion‐based 3D printing. These features enable the construction and post‐printing remodeling of tissue‐like architectures, thus offering a versatile platform for dynamic biofabrication. Notably, the regulation of cell migration in our system is governed by dynamic microporous materials, which are distinct from previously reported strategies using bulk hydrogels with nanoporous structures. These unique features offer broader applications beyond bioprinting, including spatiotemporally controlled cell delivery and tissue regeneration therapies.

Current 4D biofabrication techniques using engineered biomaterials have largely focused on the use of shape‐morphing materials or spontaneous cell‐material interactions (e.g., contraction). For example, shape‐morphing tissue constructs have been fabricated through spontaneous cellular contractions mediated through hydrogel degradation,^[^ ^50^ ^]^ the deformation of self‐assembled fibrous materials,^[^ ^84^ ^]^ or with designer materials that change properties on‐demand by stimuli, irrespective of cellular behavior.^[^ ^40^ ^]^ In contrast to these examples, our system using dynamic microgels is a unique approach that actively changes the interactions at the cell‐material interface to guide cell outcomes in a highly programmable manner, setting the stage for use across many cell and tissue systems and applications ranging from in vitro models to therapeutic tissues.

Although the current study focuses on cellular migration as a proof‐of‐principle, future work will investigate additional biological processes such as proliferation, differentiation, and matrix deposition. By enabling spatiotemporal regulation of these cellular behaviors, this approach has the potential to mimic more complicated, heterogeneous tissue structures over time. In addition, multi‐component systems using multiple types of microgels or spheroids will be explored, which would allow for more sophisticated regulation, such as local, directional control of biological processes and the formation of multicellular constructs. We believe that the principles demonstrated here are broadly generalizable to other stimuli‐responsive systems, including the use of any guest‐host pairs or alternate guest molecules with cyclodextrin in this system, and cell types. Our current work represents an important step toward the design of advanced dynamic materials capable of guiding tissue morphogenesis and function.

## 4 Experimental Section

### Materials and Instruments

Unless otherwise stated, all commercial reagents were used as received. Sodium hyaluronate (HA, mol. wt. = 70 kDa) was purchased from Lifecore Biomedical (Chaska, MN), lithium phenyl‐2,4,6‐trimethylbenzoylphosphinate (LAP) was purchased from Colorado Photopolymer Solutions (Boulder, CO), and tetra‐PEG20k‐thiol was purchased from Creative PEGWorks (Chapel Hill, NC). Dulbecco's phosphate‐buffered saline (PBS) was purchased from Gibco. PBS or culture media containing Ad‐COOH were prepared by adding 1‐Adamantanecarboxylic acid (Sigma, #106399) powder in PBS or culture media, followed by adjusting pH with NaOH aqueous solution to 7.4 and stirring until the powder fully dissolved. Unless otherwise specified, all other reagents were purchased from Sigma–Aldrich, Fisher Scientific, and Tokyo Chemical Industry. ^1^H‐NMR spectra were obtained on a Bruker Avance III (400 MHz) with tetramethylsilane (0 ppm) as the internal standard. Photographs and movies were taken by an iPhone 14 (Apple).

### Synthesis of NorHA

Norbornene‐modified HA (NorHA) was synthesized as previously described.^[^ ^49^ ^]^ Briefly, sodium HA was dissolved in DI (deionized) water and protonated with AmberChrom proton exchange resin (50Wx8, 100–200 mesh, 3:1 ratio, resin:HA) for 2 h, titrated to pH 7.02–7.05 with tetrabutylammonium (TBA) hydroxide, frozen, and lyophilized to form HA‐TBA. HA:TBA ratio was determined to be 1.77 by the methyl peak on TBA (δ≈2.0–1.8, 12H) normalized to the methyl peak (δ≈0.7–0.9, 3H) on HA via ^1^H‐NMR (Figure S16a, Supporting Information). HA‐TBA was modified with norbornenes through di‐tert‐butyl decarbonate (Boc_2_O) coupling. Briefly, 5‐norbornene‐carboxylic acid (3 eq), 4‐dimethylaminopyridine (3 eq), and Boc_2_O (1 eq) were added to an anhydrous dimethyl sulfoxide (DMSO) solution of HA‐TBA (1 eq) and allowed to react for 24 h under N_2_. The reaction was quenched with cold DI water and then placed on dialysis (6–8 kDa molecular weight cutoff) for 2 days with DI water and NaCl salt. The aqueous solution was then precipitated in acetone, followed by a subsequent 3 days with DI water, frozen, and lyophilized. The extent of norbornene modification was determined via ^1^H‐NMR to be ≈84% (δ≈5.8–6.2, 2H) of the disaccharide repeat units of HA when normalized to the methyl peak on HA (Figure S16b, Supporting Information). The solubility of NorHA was also investigated to show that NorHA has comparable solubility to the original HA (Figure S17, Supporting Information). To further compare the hydrophobicity of NorHA, the LogD values were calculated at physiological pH (7.4) using cLogP values generated by ChemDraw and the reported pKa of HA (≈3.0),^[^ ^85^ ^]^ applying the equation: Log D = cLogP−Log(1+10 ^pH−pKa^). The calculated LogD values were −9.54 for HA and −7.91 for NorHA, indicating strong hydrophilicity even after modification.

### Microgel Fabrication and Size Characterization

AdR and NR microgels were fabricated via a water‐in‐oil emulsion. Mono(6‐mercapto‐6‐deoxy)‐beta‐cyclodextrin (BOC science, CD‐SH, 100 mM) and 1‐Adamantanethiol (Ad‐SH, 100 mM) were dissolved in DMSO to form a stock solution. The NorHA precursor solution was made by dissolving NorHA in PBS with 0.1 wt.% LAP and mixing with the DMSO stock of CD‐SH and Ad‐SH. To form an emulsion, the precursor solution was added to the oil phase (98% light mineral oil/ 2% Span 80) dropwise, allowed to stir (560 rpm) for 30 sec., and crosslinked with light (320–390 nm, Omnicure lamp, 20 mW cm^−2^) for 30 min. To prepare NR microgels, DTT was used instead of CD‐SH and Ad‐SH (4 mM DTT and 560 rpm for Soft NR microgel; 8 mM DTT and 280 rpm for Stiff NR microgel) (Figure S3a, Supporting Information). Microgels were washed by discarding the oil phase, soaking in a solution of 1% tween 20 in PBS, and washing with PBS five times. AdR microgels were placed in 5 mM Ad‐COOH solution overnight, followed by washing out with PBS five times to remove unreacted CD‐SH and Ad‐SH. Microgels were stored at 4 °C before use in experiments. Fluorescein isothiocyanate (FITC)‐dextran (0.2 wt.%, MW: 2 million Da) was added into the NorHA precursor solution to allow visualization of microgels for size characterization. To allow for cell adhesion, 2 mM RGD peptide (GenScript, #SC1208, sequence: GCGYGRGDSPG) was added into the NorHA precursor solution and covalently incorporated during the microgel fabrication via thiol‐ene reaction between the thiol group of the cysteine residue in the peptide and the norbornene groups on NorHA. Microgels were visualized with a wide‐field fluorescent microscope, suspended in PBS solution alone or with varied concentrations of Ad‐COOH for 30 min before measuring diameters. To wash out Ad‐COOH, microgels were centrifuged (15 000 g), and the supernatant was discarded and replaced with PBS, which was repeated 3 times. For kinetic studies, the suspension of microgels was mixed with the same volume of 5 mM Ad‐COOH solution, and imaging was performed at various times. The plots of concentration‐dependent and time‐dependent diameter changes were fit to a one‐phase association curve by GraphPad Prism 9 (Dotmatics).

### Microgel Nanoindentation

All nanoindentation measurements were performed on a FemtoTools FT‐MTA03 nanomechanical tester with a 25 µm radius glass spherical indenter probe (FT‐S2’000) as previously described with slight modification.^[^ ^86^ ^]^ Briefly, all microgels were stained with 1:1000 Alcian blue solution for 20 s, washed, submerged in PBS for hydration during testing, and aligned with the spherical indenter probe using a 90° brightfield microscope camera. For Ad‐COOH (+) microgel conditions, 5 mM Ad‐COOH in PBS was added to the microgels and incubated for 2 h before indentation testing. To reduce drag force noise, microgels were approached at 0.1–0.35 µm s^−1^ until triggered at a max force of 0.1 µN. Once triggered, the microgels were loaded to a max of 5 µm at a load rate of 1 µm s^−1^ and an unload rate of 2.5 µm s^−1^. All microgels were tested at the highest point to ensure that the proper contact radius was calculated to determine the reduced modulus (E_r_). The Oliver‐Pharr equation E_r_ = (sqrt(π)/2ß)*(S/sqrt(A)), where S: contact stiffness, ß = 1 for spherical tips, and A: contact area, was used to determine the E_r_ for each microgel. Microgel contact area was determined via indenter‐particle‐substrate method as previously described,^[^ ^67^ ^]^ where A = π*(2*a_particle_*a_substrate_/a_particle_+a_substrate_)2 and a: contact radius. Energy loss (pJ) was determined by subtracting the area under the force‐displacement loading curve from the area of the unloading curve above the x‐axis.

### Granular Hydrogel Formation, Post‐Crosslinking, and Characterization

Granular hydrogels were prepared by centrifuging microgels (15 000 g, 5 min) in microcentrifuge tubes and removal of the supernatant. For post‐crosslinking, granular hydrogels were re‐suspended in PBS containing tetra‐PEG20k‐thiol solution (0.1 wt.% for AdR granular hydrogels and 0.5 wt.% for NR granular hydrogels) and LAP (0.05 wt.%), followed by centrifuging (15 000 g, 5 min) to produce granular hydrogels containing the crosslinker. The AdR hydrogels incorporated dynamic host–guest interactions (Ad–CD), which provide cohesive interparticle interactions even without covalent post‐crosslinking. In contrast, the NR hydrogels do not contain such supramolecular bonds, so a higher concentration was used to match the mechanical properties of the AdR system (Figure S18, Supporting Information). The granular hydrogels with tetra‐PEG20k‐thiol were then transferred to disc‐shaped PDMS molds (d = 8 mm, h = 1.5 mm) by a spatula and exposed to visible light (400–500 nm, Omnicure, 20 mW cm^−2^) for 5 min. The post‐crosslinked hydrogels were soaked in PBS overnight before use. The hydrogels were incubated with PBS containing a specific concentration of Ad‐COOH for 30 min before measuring their diameter. For washing out conditions, the solution was removed and replaced with fresh PBS (ca. 50x volume), incubated for 10 min, and this process was repeated at least three times. For kinetics studies, hydrogels were transferred to 5 mM Ad‐COOH in PBS immediately before measuring diameter changes at different times. The plots of concentration‐dependent and time‐dependent diameter changes were fit to a one‐phase association curve.

To prepare stiff NR microgels with different packing densities, five different conditions were used (Figure S10, Supporting Information). To obtain highly jammed granular hydrogels, a slurry of microgels containing tetra‐PEG20k‐thiol solution (0.5 wt.%) and LAP (0.05 wt.%) was filtered under vacuum through pluriStrainer (pluriSelect, mesh size: 40 µm). For intermediately jammed granular hydrogels, NR microgels were centrifuged at 15 000 or 500 g for 5 min in microcentrifuge tubes, followed by removal of the supernatant. To obtain granular hydrogels with lower packing densities, stiff NR granular hydrogels after centrifuging (15 000 g, 5 min) were diluted with PBS containing tetra‐PEG‐thiol (0.5 wt.%) and LAP (0.05 wt.%) to obtain 80 or 60 vol% concentrations. These hydrogels were then transferred to a 96 well plate and mixed with bMSC spheroids, followed by post‐crosslinking.

### Confocal Imaging of Microporous Structures

Post‐crosslinked granular hydrogels were prepared in disc‐shaped PDMS molds (d = 6 mm, h = 1.5 mm) and transferred to a 96‐well plate. TMR‐dextran (0.1 wt.%, 500 kDa) in PBS was added, followed by incubation at room temperature for more than 2 h. A confocal microscope (Nikon, Eclipse Ti2) equipped with a 10× objective lens was used to visualize TMR‐dextran within the interstitial pores. Before imaging, the hydrogels were placed in PBS containing various concentrations of Ad‐COOH and TMR‐dextran (0.1 wt.%, 50 kDa) for 30 min. Multiple 3D stacks with average z‐depths of 200 µm and interslice spacing of 5 µm were acquired at randomly selected regions of interest (ROIs). Imaging data were further analyzed using FIJI as previously reported.^[^ ^69^ ^]^ Fluorescent signals at each slice were thresholded, smoothed, and the area fraction of the fluorescent signal was obtained using the in‐built Analyze Particles function (particles with an area of >50 µm^2^ were counted to remove noise). The mean particle area and the percentile area occupied by the fluorescent signal were defined as average pore size and porosity, respectively. Imaris software was used to visualize 3D porosity of the granular hydrogels using the same raw confocal datasets. For washing out conditions, the solution was removed and replaced with fresh PBS (ca. 50x volume) containing TMR‐dextran, incubated for 10 min, and this process was repeated at least three times. For kinetics studies, hydrogels were transferred to a solution of 5 mM Ad‐COOH and TMR‐dextran in PBS before real‐time imaging. For the confined conditions, the granular hydrogels containing tetra‐PEG20k‐thiol solution (0.5 wt.%) and LAP (0.05 wt.%) were transferred to a 96 well plate and then post‐crosslinked with visible light (20 mW cm^−2^, 5 min). D‐PBS with/without 5 mM Ad‐COOH was added to the wells. The plots of concentration‐dependent and time‐dependent diameter changes were fit to a one‐phase association curve.

### Microwell Fabrication

Microwell pyramid arrays were fabricated from polydimethylsiloxane (PDMS) as previously reported.^[^ ^48^ ^]^ Briefly, PDMS (polymer:crosslinker ratio = 10:1) was degassed, poured into a commercial microwell plate (AggreWell 400, StemCell Technologies), and cured at 80 °C for 2 h. The resulting PDMS was then treated with trichloro(1H,1H,2H,2H‐perfluorooctyl)silane after O_2_ plasma activation to produce a negative mold. Positive molds were created by adding PDMS precursors (polymer:crosslinker ratio = 10:1) onto the negative PDMS molds in 6 well plates, degassing, and curing at 80 °C for 2 h. The positive molds were soaked in isopropyl alcohol for 1 h, rinsed in DI water overnight, and sterilized under a UV germicidal tissue culture hood for at least 30 mins. Each well was rinsed with PBS, and an anti‐adherence solution (Stem Cell Technologies) was added. Plates were centrifuged at 1000 x g for 5 mins to remove air bubbles. Finally, the wells were aspirated and filled with culture medium until use.

### Cell Isolation and Expansion

Mesenchymal stromal cells (MSCs) were isolated from bone marrow obtained from juvenile bovine femurs (Research 87, Boylston, MA) as previously described.^[^ ^87^ ^]^ Briefly, bovine joint capsules were opened in a tissue culture hood using #22 surgical blades and tweezers. Menisci and excess synovial tissue were removed to access the femur cartilage and bone layers. Using a 300 mm bi‐metal hacksaw, the articular cartilage layer was removed, and trabecular bone was collected in 50 mL conical tubes. PBS was added to the trabecular bone, shaken to disrupt the bone marrow from the pores, and poured into new conical tubes. This process was repeated, supernatant centrifuged at 500 x g for 5 min, and the cell pellet resuspended in dissection media comprised of Dulbecco's modified Eagle Medium, 1% Penicillin/Streptomycin (P/S), and 1% Amphotericin B. Cell suspensions were plated in 10 cm tissue culture plates and expanded to confluency before passaging and shifting to expansion media. Bovine MSCs (bMSCs) were expanded in α‐modified Eagle's medium (α‐MEM), 10% fetal bovine serum (FBS), 1% P/S, and basic fibroblast growth factor (bFGF, 1 ng mL^−1^).

### Spheroid Preparation

MSCs were passaged to P3. For spheroid preparation, bMSCs were trypsinized and resuspended in culture medium. 1000 cells per well within the PDMS molds were seeded, and the PDMS molds were placed in 6 well plates with 5 mL of α‐MEM containing 10% FBS, 1% P/S, bFGF, 50 µg mL^−1^ ascorbic acid. Cells were incubated for 48 h under 5% CO_2_ to form spheroids.

### Preparation of Microgel/Spheroid Composites

Spheroids were gently collected by pipetting and centrifuged at 300 x g for 20 sec, and the supernatant was removed to obtain a suspension containing 5–10 spheroids per µL. Microgels were sterilized under a UV germicidal tissue culture hood for at least 30 min. RGD‐modified microgels were centrifuged (15 000 x g, 5 min) with the post‐crosslinker, and transferred to 3D printed disc‐shaped molds (PLA, d = 4 mm, h = 2 mm, ca. 25 µL). 2 µL spheroid suspension was mixed with hydrogels, followed by exposure to visible light (400–500 nm, Omnicure, 20 mW cm^−2^) for 5 min. The composite was placed in α‐MEM (+ 10% FBS, + 1% P/S, + bFGF, +/– 5 mM Ad‐COOH) and incubated for 2, 4, or 6 days under 5% CO_2_ to allow cell migration. For the control experiment using bulk hydrogels, the precursor solution of NorHA/Ad‐SH/CD‐SH/LAP containing bMSC spheroids (10 spheroids per µL) and 2 mM RGD‐peptide was prepared and photo‐crosslinked with visible light (20 mW cm^−2^, 5 min). For dynamic culture conditions, the media containing Ad‐COOH was removed, replaced with fresh Ad‐COOH free media (ca. 50x volume), incubated for 15 min, and this process was repeated at least three times. At specific time points, the composites were rinsed with PBS and fixed under 7.5% formalin without/with 5 mM‐Ad‐COOH for at least overnight.

### Cell Staining and Migration Assay

The fixed composites were rinsed with PBS 5 times. Bovine serum albumin (BSA) solution (3 wt.%) containing 0.1% Triton‐X was added into each well, and placed for 2 h at room temperature (blocking). After that, the fixed composites were rinsed with PBS 5 times, stained with Phalloidin‐Alexa647 (Invitrogen,#A22287) in the presence of 1 wt.% BSA and 0.1% Triton‐X for 2 h at room temperature, washed with PBS 5 times, and imaging was performed. For Ad‐COOH containing conditions, PBS containing 5 mM Ad‐COOH was used. A confocal microscope equipped with a 10× objective lens was used to visualize spheroids and microgels. Individual spheroids were randomly selected as regions of interest (ROIs). Multiple 3D image stacks were acquired, each spanning ≈200 µm above and below the spheroid center, with an interslice z‐spacing of 10 µm. To analyze migration distance, z‐projected images were created from z‐stacked images, and the radius of Alexa647 fluorescence was quantified. The maximum distance could be underestimated as well if cells were migrating completely perpendicular to the MSC spheroid, which was not accounted for during projection, as it overlays within the MSC spheroid. Regardless, it was believed that this analysis gives an accurate depiction of the differences between groups.

A confocal microscope equipped with a 2x objective or 10x lens was used to visualize printed spheroids. To obtain 3D images of printed spheroids, the tissue clearing reagent, RapiClear 1.52 (SUNJin lab, #RC152001) was used. After the same staining conditions were conducted with 2% Triton‐X, the samples were immersed in the RapiClear solution at 4 °C overnight and then imaged. 3D surface‐rendered images were reconstructed from z‐stacked images by Imaris. For spheroid rendering, 2‐µm surface grain size smoothing and 100µm‐largest‐diameter sphere background elimination were used. For microgel, 5‐µm surface grain size smoothing and absolute intensity thresholding were used.

### Rheological Experiments

The rheological properties were measured by an oscillatory shear rheometer (TA Discovery HR‐20, TA Instruments) equipped with an 8 mm parallel plate geometry set to a 1500 µm or 800 µm gap. Granular hydrogels were transferred by a spatula to the plate to fill the geometry. For flow characterization, viscosity was measured with a continuously ramped shear rate (from 0 to 100 s^−1^). Strain sweeps (1−100% strain, 1 rad s^−1^) were used to assess shear‐yielding properties. For shear recovery experiments, low (1%) and high (100%) strains were periodically applied at a frequency of 1 rad s^−1^. All tests were conducted at room temperature.

### Suspension Bath Printing

A custom‐built 3D printer modified from Original Prusa MK4 was used for 3D printing (Figure S9, Supporting Information for the details). An Original Prusa MK4S was modified with two custom syringe extruders, enabling the precise spatiotemporal deposition of fluid materials. A Prusa MK4S was purchased as a kit, assembled, and calibrated with thermoplastic filaments. The extruder mechanism and control board were replaced with two custom syringe extruders and a Duet 3 Mini 5+ control board. The highly versatile syringe extruders represent the latest combined evolution of low‐cost, high‐precision bioprinting attachments developed by the Feinberg Lab, known as the “Replistruder”,^[^ ^30^, ^88^ ^]^ and the Crosby Lab (the “Enderstruder”).^[^ ^89^ ^]^ Briefly, the main structural components were 3D‐printed from PETG filament on the Original Prusa MK4S, and the remaining components, including the central rod, bearings, pulley‐and‐gear system, and fasteners, were purchased from Amazon. A NEMA 17 stepper motor (400 microsteps) was attached to a 20‐tooth gear that drives a 40‐tooth gear via a 180 mm curved‐tooth timing belt. The pitch of the central rod, threaded through the moving carriage, was given as 2 mm, resulting in a theoretical displacement of 20.76 nL per step. A custom sys file was created in RepRapFirmware Configuration Tool; the e‐steps value was set to 800 steps mm^−1^ (never to be changed). A custom profile was created in PrusaSlicer, and the “filament” diameter was set to 2.3 mm, which represents the diameter of the 250 µL Hamilton gastight syringe (# 81120). Extrusion multiplier of 6 was used. 3D geometries were created in Autodesk Fusion, sliced in PrusaSlicer, and then sent to the Duet via Ethernet.

Rectangular molds (8 mm x 6 mm x 1.5 mm) for suspension bath printing were made using a Bambu Lab A1 printer using PLA filaments. Prusa Slicer was used to design the printing patterns and control the 3D printer. For printing, an extrusion multiplier of 16 and 2 mm s^−1^ for needle speed were used, and the extrusion rate was determined to be 0.78 µL s^−1^. bMSC spheroids (>200000) were jammed at 300 x g for 20s on ice, and then the supernatant was removed. The obtained pellet was transferred into a 250 µL Hamilton syringe with a 22G needle (Inner diameter: 0.41 mm). The syringe was inverted to further jam spheroids by gravity, and the remaining liquid fraction was extruded. Granular hydrogels swollen with 5 mM Ad‐COOH, in the presence of tetra‐PEG20k‐thiol and LAP, were prepared and loaded into a 3D printed mold using a spatula. The spheroid ink was primed until stable and continuous lines were obtained. After printing, the objects were exposed to visible light (Omnicure lamp, 400–500 nm, 20 mW cm^−2^, 5 min) and the printed structures were placed in culture medium. At specific time points, the objects were washed with culture media three times, and then fixed and stained as described above. All fabrication processes and imaging procedures were performed at room temperature. For quantitative analysis, the widths of printed lines at 15 different sections were measured in images using the Huang threshold method. The values were averaged to obtain the mean line widths.

### Statistical Analysis

All statistical tests were performed in GraphPad Prism 9 (Dotmatics). For comparisons between two groups, Welch's t‐test was completed (significance determined by p < 0.05). For comparisons among multiple groups, either ordinary one‐way ANOVA with Tukey's multiple comparisons post hoc test or, when variance assumptions were not met, Welch's ANOVA or Brown–Forsythe ANOVA test was used. Homogeneity of variance was evaluated using Bartlett's and Brown–Forsythe tests, as appropriate. Bar graph descriptors (mean ± standard deviation) are displayed within the figure panel caption. Experimental *n* values are stated in figure captions.

## Conflict of Interest

The authors declare no conflict of interest.

## Supporting information



Supporting Information

Supplemental Movie 1

Supplemental Movie 2

Supplemental Movie 3

## Data Availability

The data that support the findings of this study are available from the corresponding author upon reasonable request.
