# Supplementary material for: Dynamic Regulation of Granular Hydrogels Through Guest‐Host Interactions to Spatiotemporally Guide Cellular Migration
Source: Adv Sci (Weinh). 2025 Nov 9;13(5):e12971. doi: 10.1002/advs.202512971 (PMC12850067; doi:10.1002/advs.202512971)
Supplement: Supplementary file 1 — Supporting Information [file ADVS-13-e12971-s001.pdf]

Supporting Information

Dynamic Regulation of Granular Hydrogels through Guest-host Interactions to Spatiotemporally Guide Cellular Migration

Keisuke Nakamura[a], Nikolas Di Caprio[a], Jonathan T. Taasan[b], Cody O. Crosby[a,c], Jason A. Burdick[a,b]*

[a]BioFrontiers Institute, University of Colorado Boulder, Boulder, CO, 80303 USA

[b]Department of Chemical and Biological Engineering, University of Colorado Boulder, Boulder, CO, 80303 USA

[c]Department of Physics, Southwestern University, Georgetown, TX, 78626

*Correspondence: jason.burdick@colorado.edu

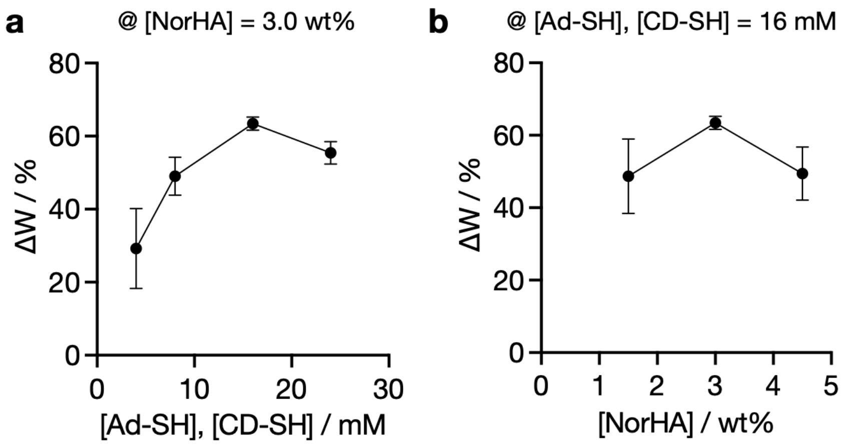

Figure S1. Optimization of the concentrations of hydrogel components. Weight changes (ΔW) with the addition of Ad-COOH to bulk hydrogels prepared with (a) varied concentrations of Ad-SH and β-CD-SH at a constant NorHA concentration (3 wt%) or (b) varied NorHA concentrations at a constant Ad-SH and β-CD-SH concentration (16 mM). $n = 3$. $\Delta W = (W_{after} - W_{before}) / W_{before}$. ($W_{after}$: The weight of the hydrogel after swelling with 20 mM Ad-COOH, W_{before}: The weight of the hydrogel before swelling). In panel a, the NorHA concentration is kept constant (~50 mM of norbornene moieties), while the concentrations of Ad–SH and CD–SH are varied. As the Ad–SH/CD–SH concentrations increase, the swelling response initially increases due to the higher density of guest–host crosslinks that can be competitively displaced by Ad–COOH. At high concentrations (e.g., 24 mM), incomplete dissolution likely results in turbidity that inhibits UV penetration during photopolymerization, thereby reducing effective crosslinking and decreasing swelling response. This leads to a maximum swelling response around 16 mM of Ad–SH/CD–SH. In panel b, the ratio of Ad–SH/CD–SH to NorHA is kept constant while increasing the NorHA concentration. Thus, higher NorHA concentrations correspond to higher absolute amounts of Ad–SH/CD–SH, reaching up to 24 mM at 4.5 wt% HA. As in panel a, solubility issues at high concentrations likely reduce crosslinking efficiency and responsiveness, resulting in a peak swelling response around 3.0 wt% NorHA.

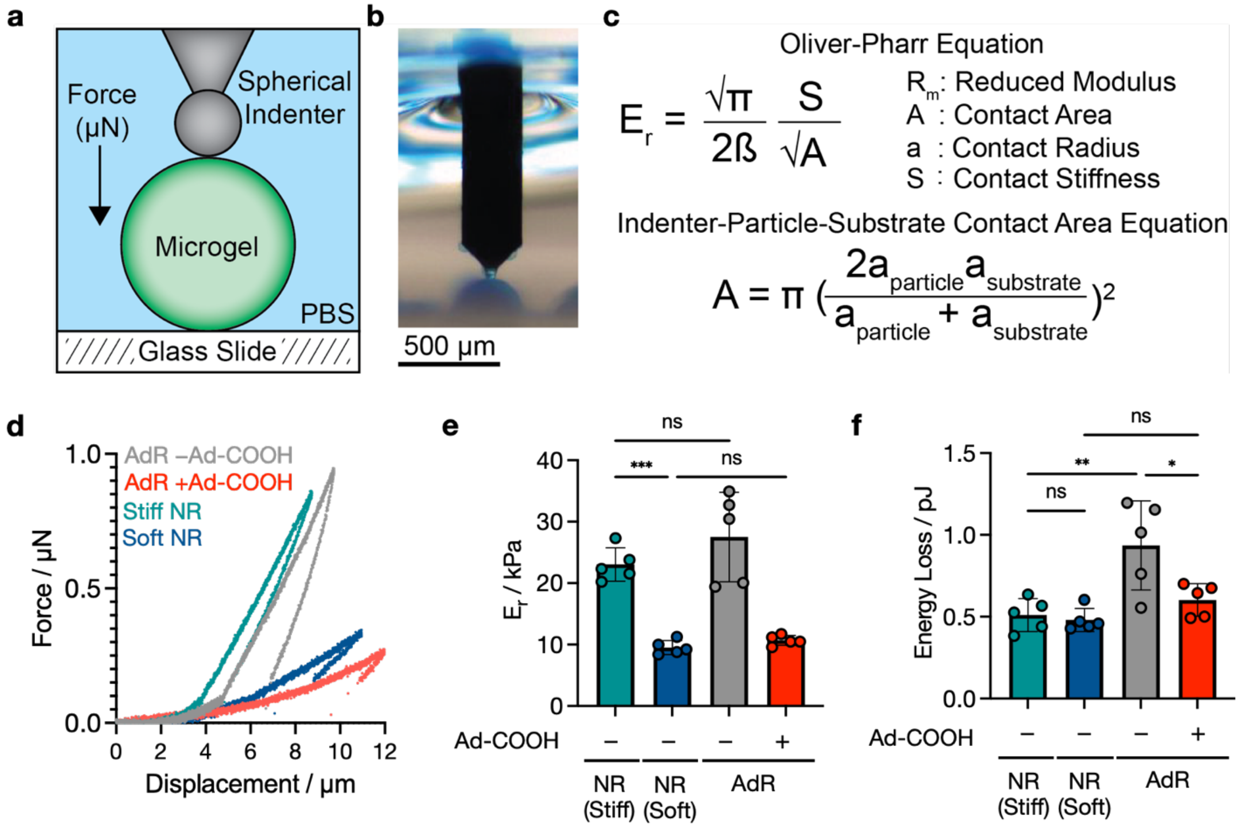

Figure S2. Nanoindentation of individual microgels. a) Schematic of experimental set up for the nanoindentation of a microgel with a spherical indenter. b) Brightfield image of the nanoindentation process for a microgel stained with Alcian blue. Scale bar: 500 μm. c) Oliver-Pharr equation to determine the reduced modulus (E_r) from nanoindentation testing. Microgel (particle on substrate) assumption used to calculate the area of the indented microgel samples with a spherical probe indenter. d) Force versus displacement curve for testing of microgels. Cyan: Stiff non-responsive (NR) microgel, Blue: Soft NR microgel, Grey: AdR microgel without Ad-COOH, Red: AdR microgel with 5 mM Ad-COOH. e) E_r calculated from the testing curves. Stiff NR and Soft NR microgels have E_r values matched with those of AdR without and with Ad-COOH, respectively. f) Energy loss of microgel determined by subtracting the area under the force-displacement loading curve from the area of the unloading curve above the x-axis. $n = 5$. $^*p < 0.05$, $^{**}\ p < 0.01$. $^{***}\ p < 0.001$, ns = not significant.

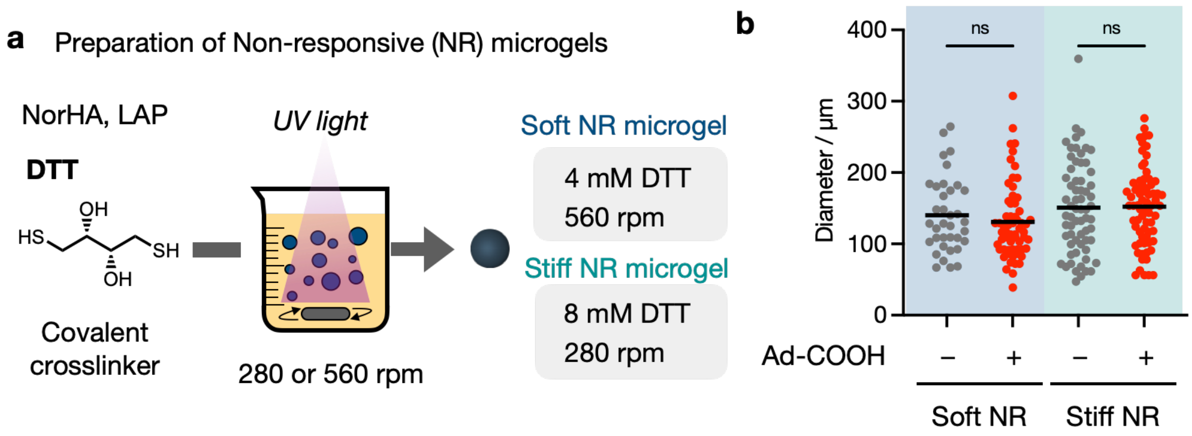

Figure S3. Preparation and characterization of non-responsive microgels. a) Non-responsive (NR) microgels are prepared through the thiol-ene reaction of NorHA (3 wt%) and DTT (4 or 8 mM) as a covalent crosslinker in the presence of LAP (0.1 wt%) and ultraviolet light. 4 mM DTT and stirring speed of 560 rpm are used for Soft NR microgels. 8 mM DTT and stirring speed of 280 rpm are used for Stiff NR microgels. b) Average diameter (n = 38 to 72) of Soft and Stiff NR microgels in the absence (Grey) and the presence (Red) of 5 mM Ad-COOH, ns = not significant.

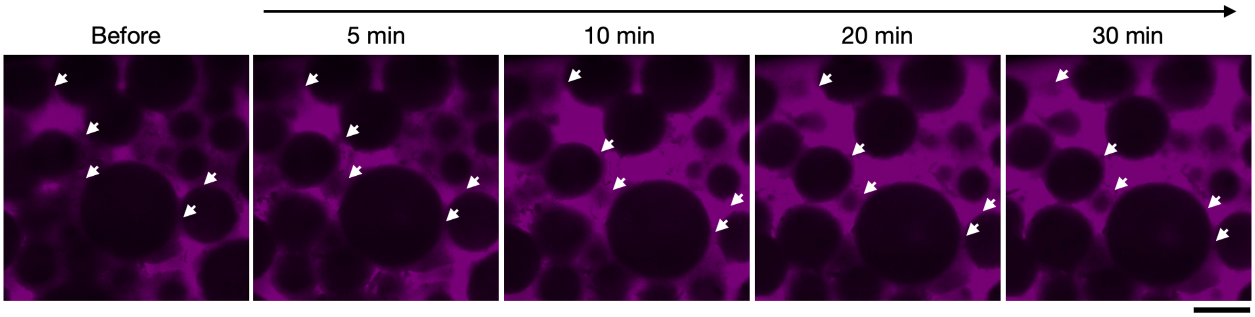

Figure S4. Time lapse of the swelling process of AdR granular hydrogels. Images acquired immediately after the addition of Ad-COOH and imaging is continued for up to 30 minutes. Arrows denote regions that suggest the loss of contact between microgels. Magenta: FITC-Dextran. Scale bar: 200 μm

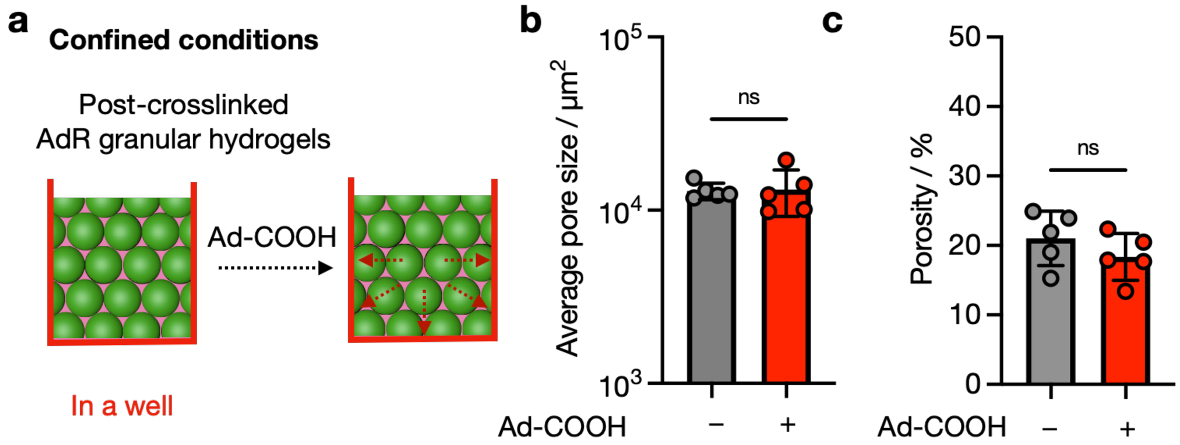

Figure S5. Microporous structures of AdR granular hydrogels in confined conditions. a) AdR granular hydrogels are post-crosslinked in a 96 plate and treated with 5 mM Ad-COOH in situ. b) Average pore sizes and c) average porosities of AdR granular hydrogels without (Grey) or when treated with (Red) 5 mM Ad-COOH for 2 hrs in the confined condition. n = 5. Data are reported as mean ± standard deviation. ns = not significant.

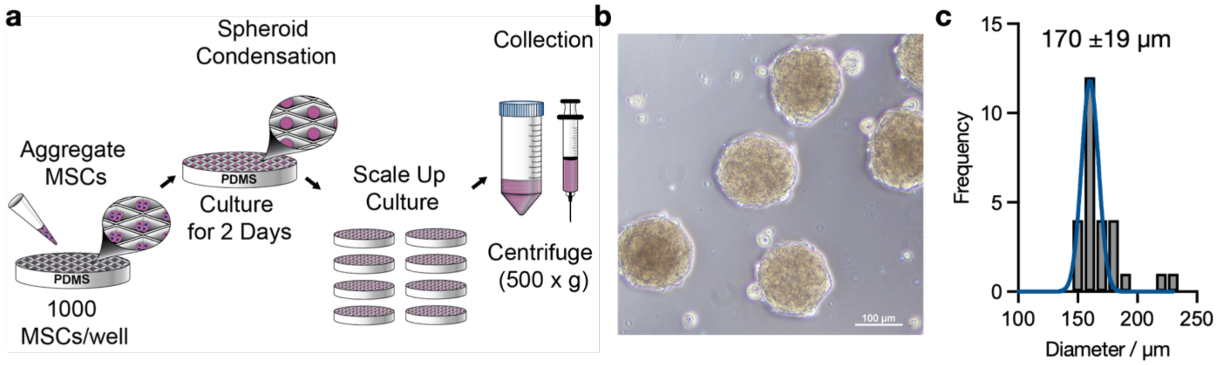

Figure S6. Preparation and characterization of bMSC spheroids. a) bMSCs (1000 per well) are seeded on PDMS molds and incubated for 2 days to condense into spheroids. b) Brightfield images of spheroids after 2 days. Scale bar: 100 µm. c) Histogram of spheroid diameter (*n* = 27). The average diameter is determined to be 170 ± 19 µm.

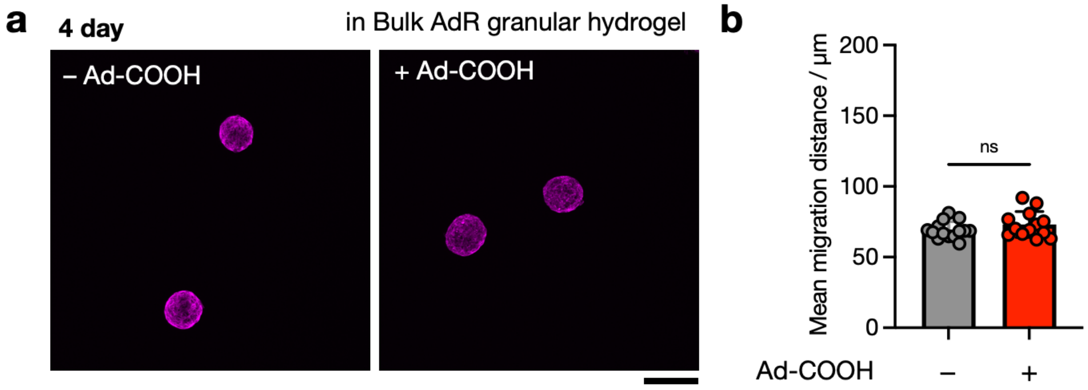

Figure S7. Migration of spheroids in bulk AdR hydrogels. a) Representative z-projected images of bMSCs spheroids without (left) and with (right) 5 mM Ad-COOH after 4 days. Magenta: spheroids (Phalloidin-Alexa647, actin). 200 µm. (b) Mean migration distance of bMSCs in bulk AdR hydrogels. n = 12 or 15. Data are reported as mean ± standard deviation. ns = not significant.

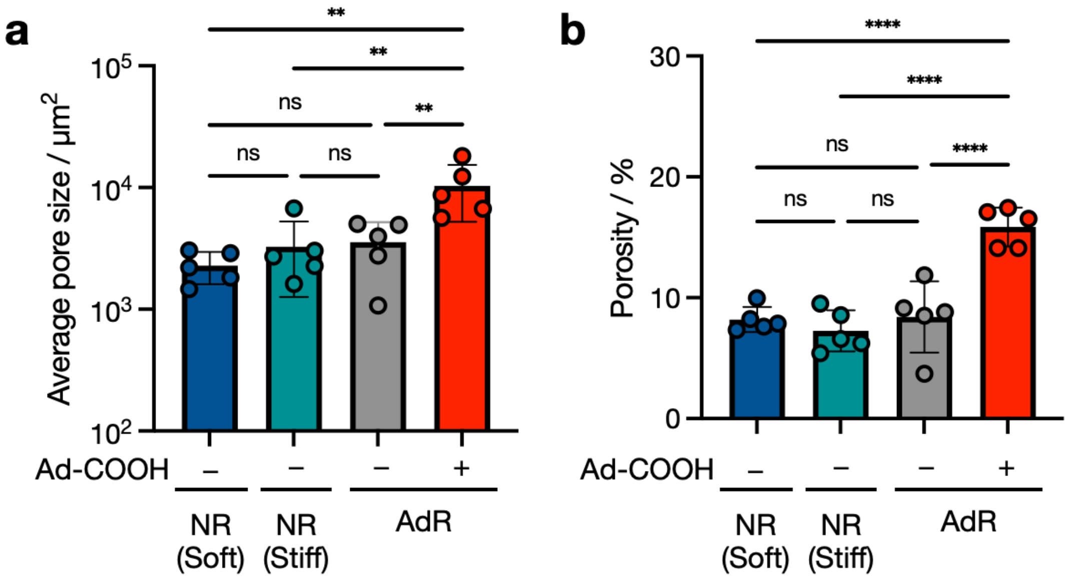

Figure S8. Comparison of microporous structure between NR granular hydrogels and AdR granular hydrogels. a) Average pore sizes (n = 5) and b) average porosity (n = 5) of granular hydrogels. Cyan: Stiff non-responsive (NR) microgel, Blue: Soft NR microgel, Grey: AdR microgel without Ad-COOH, Red: AdR microgel with 5 mM Ad-COOH. ** $p < 0.01$, **** $p < 0.0001$, ns = not significant.

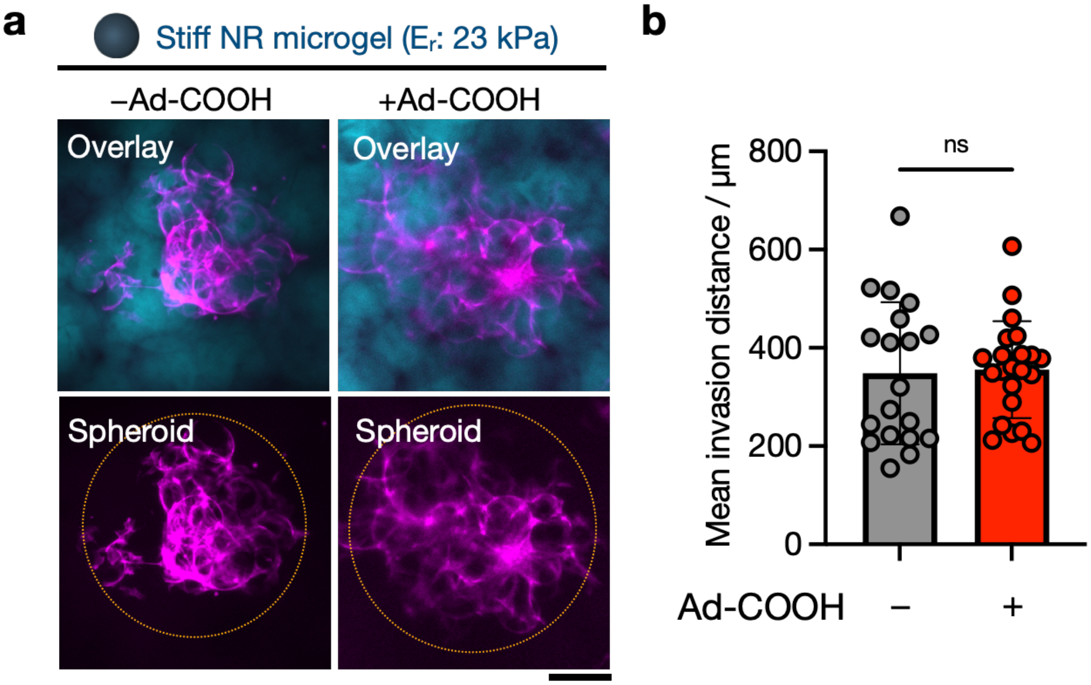

Figure S9. bMSC migration from spheroids in Stiff NR granular hydrogels with and without Ad-COOH. a) Representative z-projection confocal images and b) mean migration distance (*n* = 19 to 21) of bMSCs in AdR granular hydrogels after 4 days of culture without (left) and with (right) 5 mM Ad-COOH. Magenta: spheroids (Phalloidin-Alexa647, actin), Cyan: stiff NR microgels (FITC-Dextran). Scale bar: 200 µm, ns = not significant.

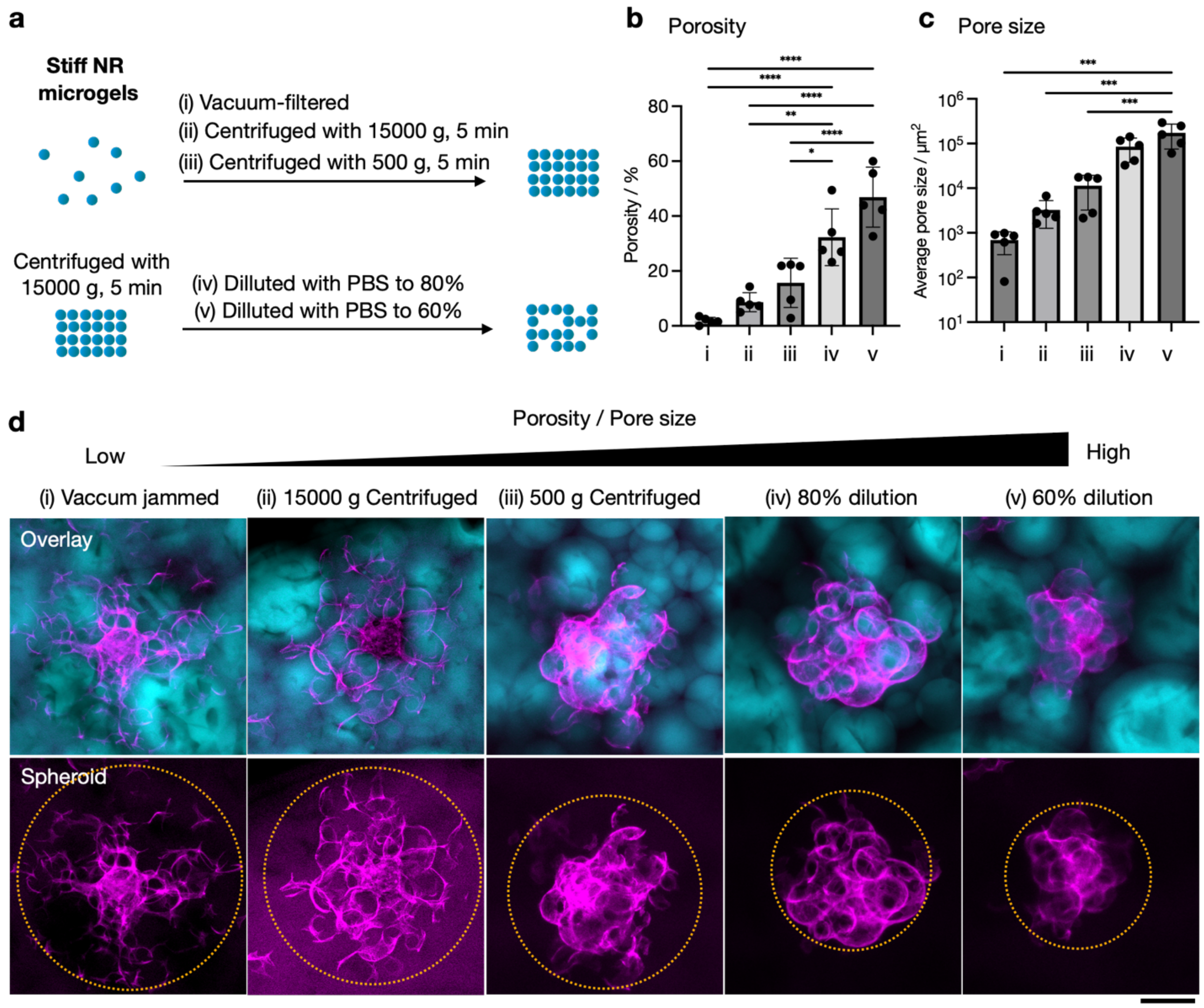

Figure S10. Preparation and characterization of NR granular hydrogels with different packing densities. a) Granular hydrogels of stiff NR microgels are prepared by vacuum filtration (i), centrifuge with 15000 x g for 5 min (ii), and centrifuge with 500 x g for 5 min (iii), or dilution to 80 % (iv) or 60% (v) with PBS after centrifuge (15000 x g, 5 min). b) average porosity and b) average pore sizes of stiff NR granular hydrogels with different jamming methods. d) Representative z-projection confocal images of bMSCs in stiff NR granular hydrogels with different extents of packing after 4 days. Magenta: spheroids (Phalloidin-Alexa647, actin), Cyan: NR microgel (FITC-Dextran). n = 5. Scale bar: 200 µm. * $p < 0.05$, ** $p < 0.01$, *** $p < 0.001$, **** $p < 0.0001$, ns = not significant.

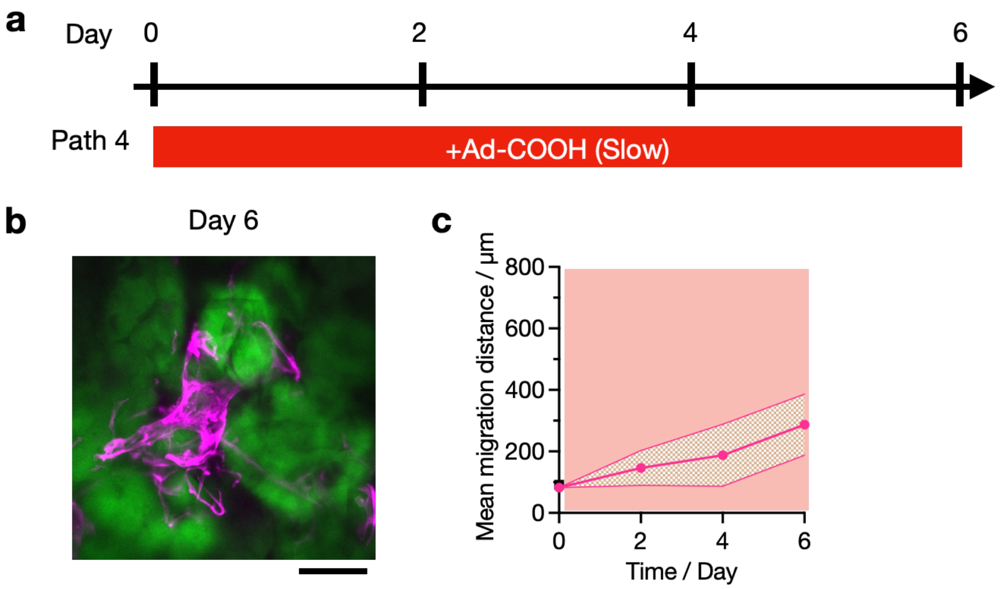

Figure S11. bMSC migration in AdR granular hydrogels with Ad-COOH. a) bMSCs were cultured in the presence of 5 mM Ad-COOH for 6 days. b) Representative z-projection images of bMSC migration from spheroids in Ad granular hydrogels at day 6. Magenta: spheroids (Phalloidin-Alexa647, actin), Green: AdR microgel (FITC-Dextran). c) Mean migration distances at different time points (0, 2, 4, 6 days). Red shading +Ad-COOH, $n = 7$ to 19. The plots at 0, 2, 4 days are the same as those shown in Figure 5d and are here extended until 6 days. Scale bar: 200 µm. Data are reported as mean ± standard deviation.

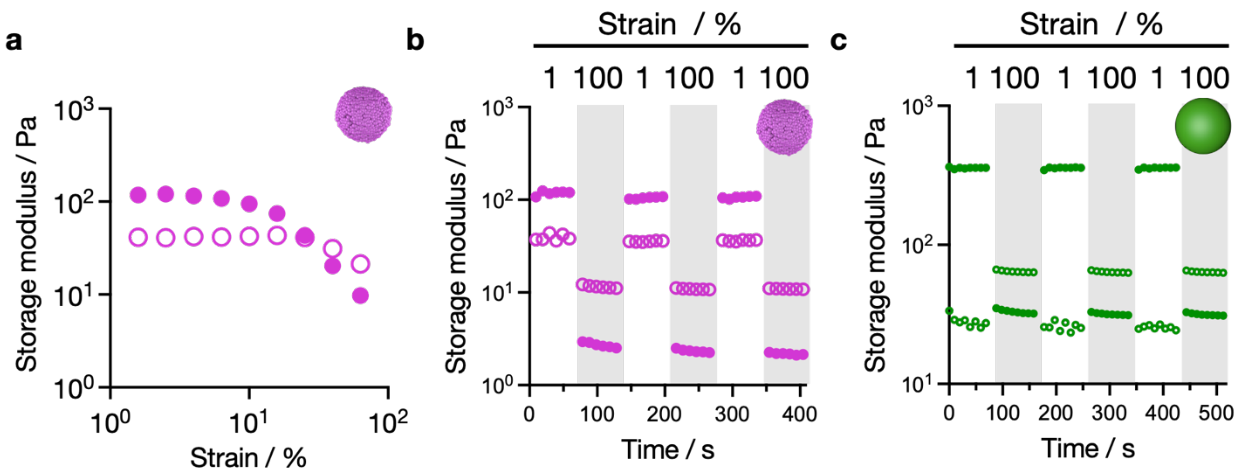

Figure S12. Rheology of jammed bMSC spheroids and AdR granular hydrogels. a) Strain sweep rheology for jammed spheroids (300 x g, 20 s). b, c) time sweep through low (1%) and high (100%) strain cycles for b) jammed spheroids and c) AdR granular hydrogels. Storage (G', filled) and loss (G'', open) moduli; Frequency: 1 rad/s.

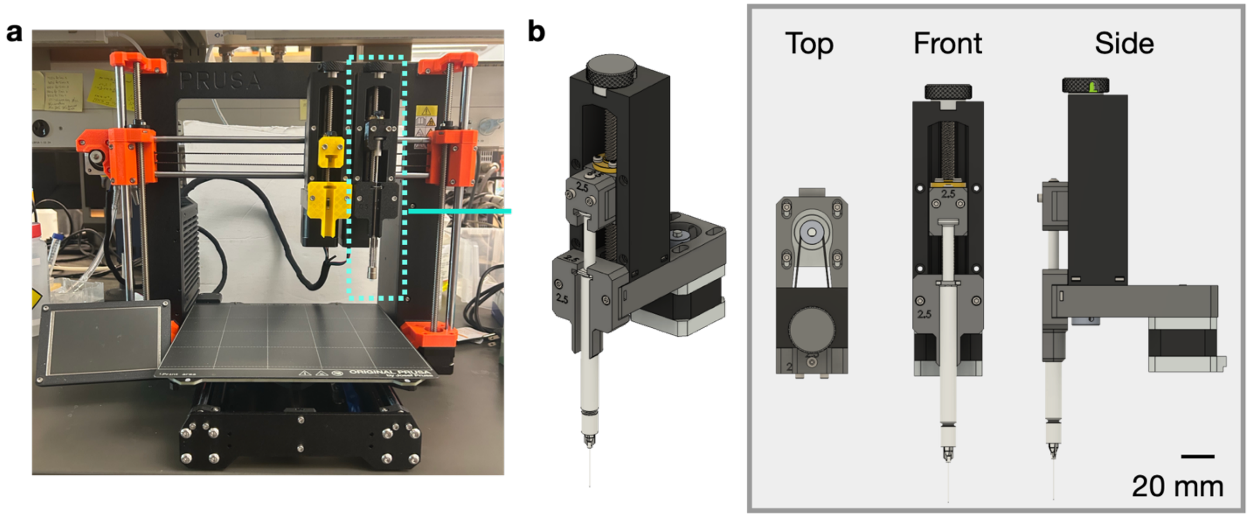

Figure S13. 3D printer design. a) Custom-built 3D bioprinter fabricated by modifying an Original Prusa MK4 with a syringe extruder. b) Syringe extruder. Design. Scale bar: 20 mm.

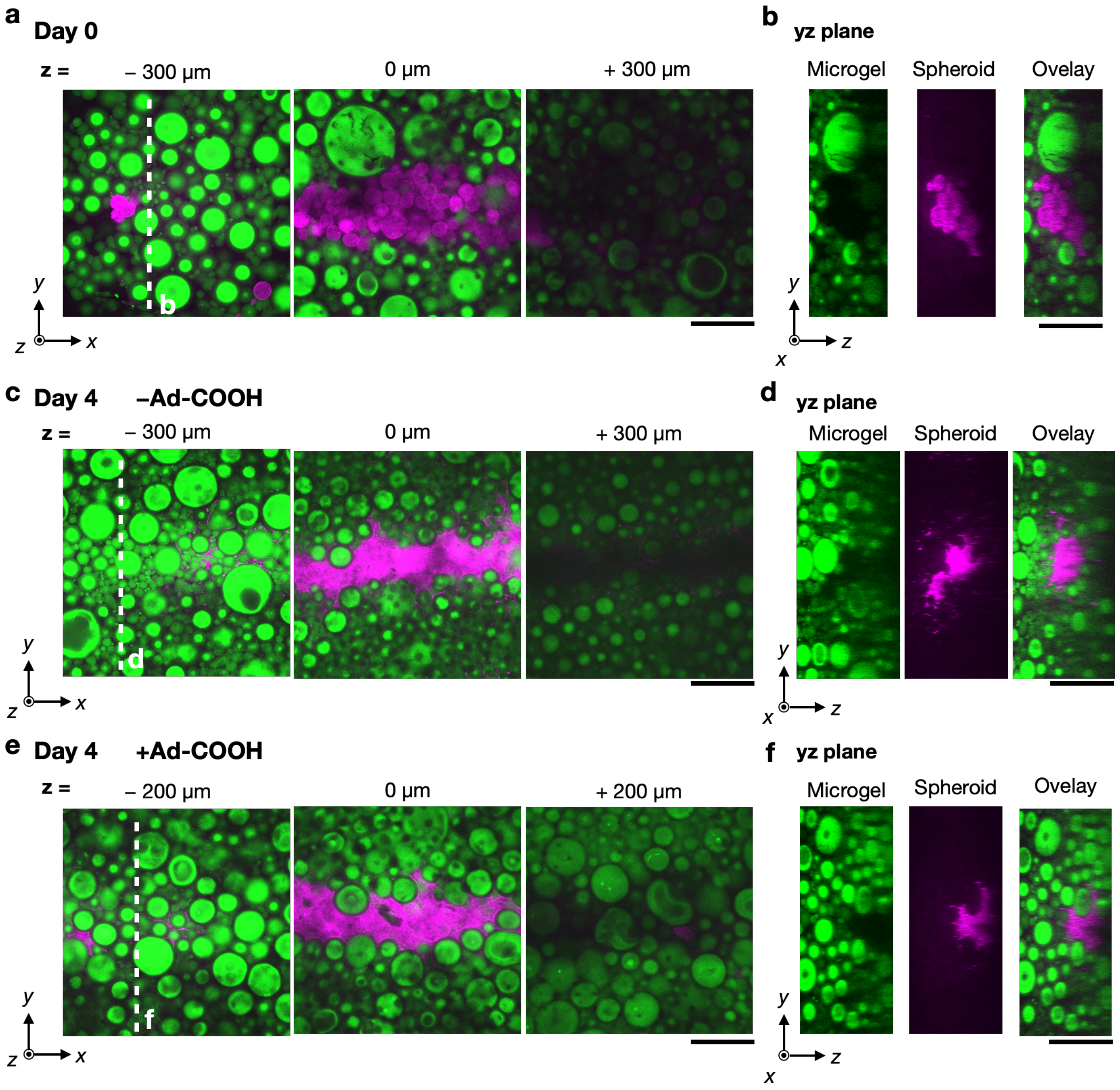

Figure S14. Confocal slice images to evaluate local distributions of microgels and spheroids. a, c, e) Confocal images at different z planes with depth into the sample (0 µm centered at middle of printed spheroid ink). b,d,f) Images along the yz plane at the intersection indicated by the dashed lines in panels a, c, e. Samples imaged at a, b) Day 0, c, d) Day 4 without Ad-COOH, e, f) Day 4 with Ad-COOH. Magenta: spheroids (Phalloidin-Alexa647, actin), Green: AdR microgel (FITC-Dextran). Scale bar: 500 µm.

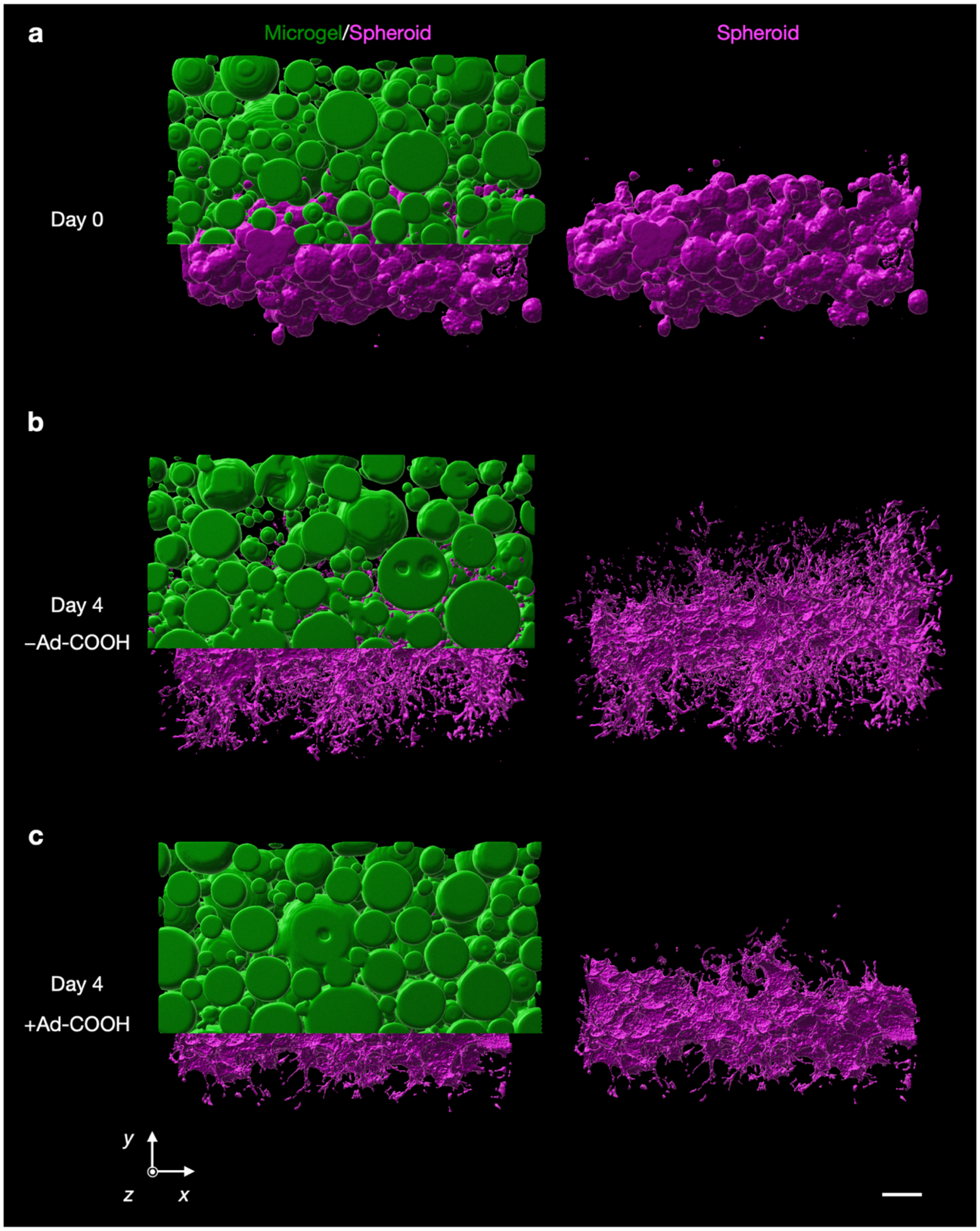

Figure S15. Surface rendered 3D images as seen from above the sample. a) Day 0, b) day 4 without Ad-COOH, c) day 4 with Ad-COOH. Left: Overlay of microgels and spheroids; Right: spheroids. Magenta: spheroids (Phalloidin-Alexa647, actin), Green: AdR microgel (FITC-Dextran). Scale bar: 200 µm.

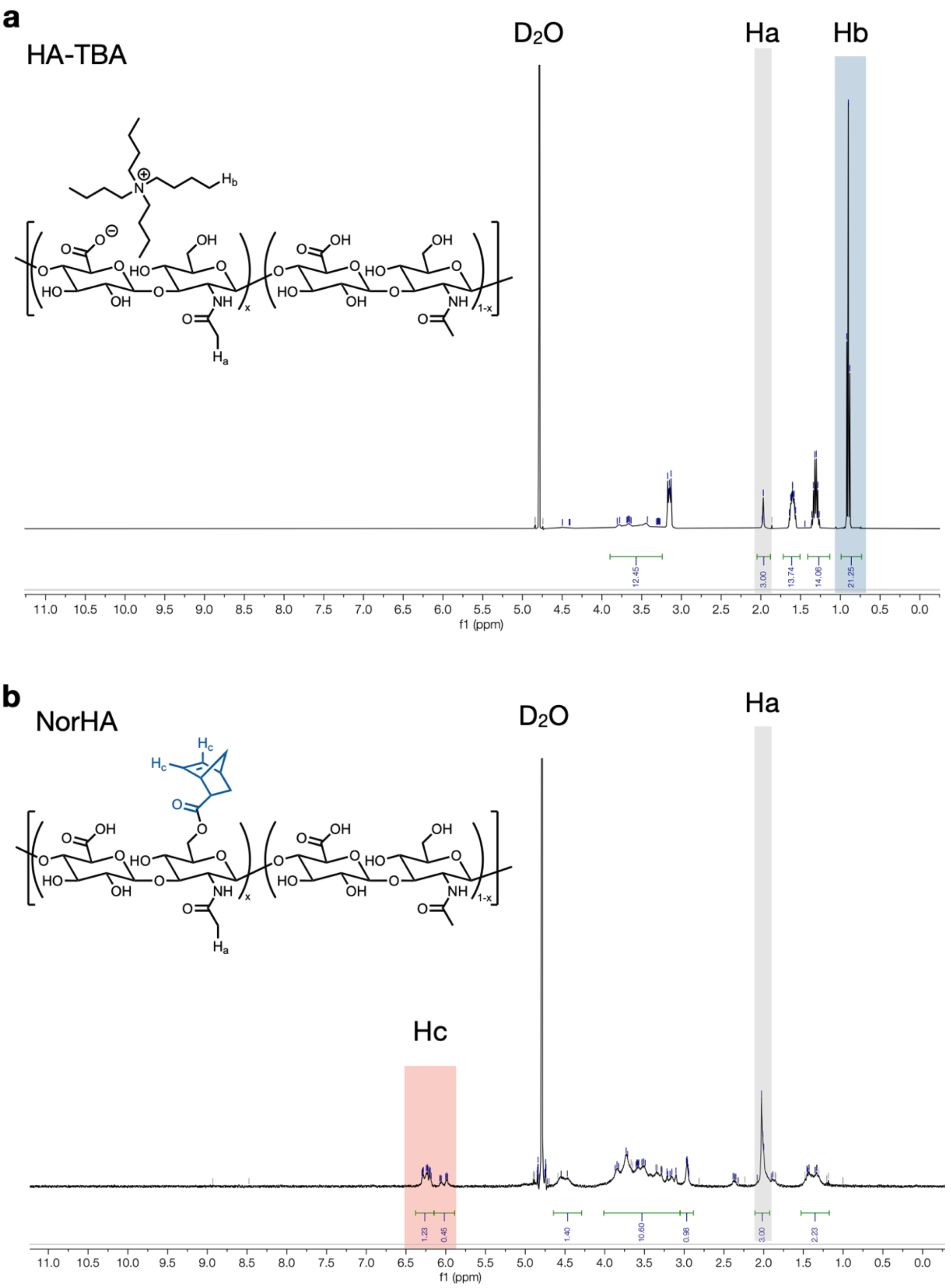

Figure S16. ^{1}H-NMR spectra of HA derivatives. a) HA-TBA and b) NorHA (400 MHz, D_2O, rt). The molar ratio of TBA over HA is determined as 1.77 based on peak areas of Ha and Hb. Modification yield of NorHA is determined to be ~84% based on Ha and Hc.

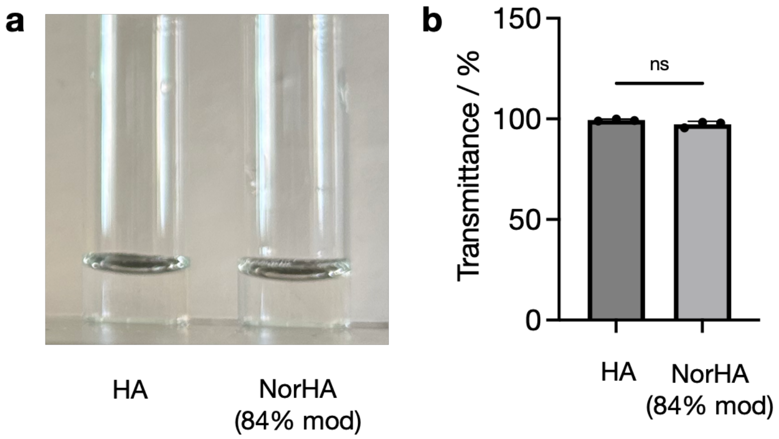

Figure S17. Solubility of Nor-HA in PBS. a) Photographs and b) solution transmittance of 3 wt% PBS solution of HA (Left) or Nor-HA (Right). These results suggest that the Nor-HA used in this study is soluble in PBS. *n* = 3.

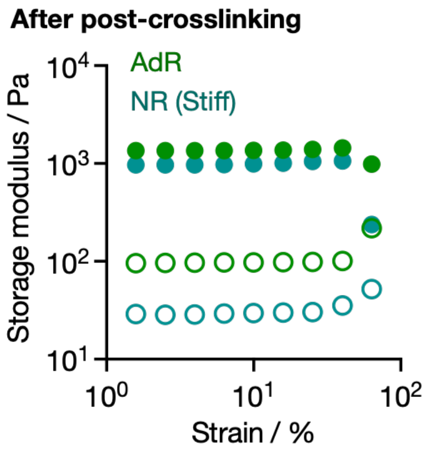

Figure S18. Storage modulus within increasing strain of post-crosslinked granular hydrogels. Strain sweeps (Frequency: 1 Rad/s, rt) of AdR granular hydrogels post-crosslinked with 0.1 mM tetra-PEG-SH (Green) and Stiff NR granular hydrogels post-crosslinked with 0.5 mM tetra-PEG-SH (Blue).

Supplementary Movies:

Movie S1. Three-dimensional surface rendered images of spheroids in an AdR granular bath immediately after printing.

Movie S2. Three-dimensional surface rendered images of spheroids in an AdR granular bath without Ad-COOH after 4 days of culture.

Movie S3. Three-dimensional surface rendered images of spheroids in an AdR granular bath with Ad-COOH after 4 days of culture.
